# Spread of aggregates after olfactory bulb injection of α-synuclein fibrils is associated with early neuronal loss and is reduced long term

**DOI:** 10.1007/s00401-017-1792-9

**Published:** 2017-12-05

**Authors:** Nolwen L. Rey, Sonia George, Jennifer A. Steiner, Zachary Madaj, Kelvin C. Luk, John Q. Trojanowski, Virginia M.-Y. Lee, Patrik Brundin

**Affiliations:** 1 Center for Neurodegenerative Science, Van Andel Research Institute, 333 Bostwick Avenue N.E., Grand Rapids, MI 49503 USA; 2 Bioinformatics and Biostatistics Core, Van Andel Research Institute, 333 Bostwick Avenue N.E., Grand Rapids, MI 49503 USA; 3 Department of Pathology and Laboratory Medicine, Institute On Aging and Center for Neurodegenerative Disease Research, University of Pennsylvania, 3600 Spruce Street, Philadelphia, PA 19104 USA

**Keywords:** Parkinson’s disease, Alpha-synuclein, Aggregates, Spreading, Neurodegeneration, Propagation, Olfactory bulb

## Abstract

Parkinson’s disease is characterized by degeneration of substantia nigra dopamine neurons and by intraneuronal aggregates, primarily composed of misfolded α-synuclein. The α-synuclein aggregates in Parkinson’s patients are suggested to first appear in the olfactory bulb and enteric nerves and then propagate, following a stereotypic pattern, via neural pathways to numerous regions across the brain. We recently demonstrated that after injection of either mouse or human α-synuclein fibrils into the olfactory bulb of wild-type mice, α-synuclein fibrils recruited endogenous α-synuclein into pathological aggregates that spread transneuronally to over 40 other brain regions and subregions, over 12 months. We previously reported the progressive spreading of α-synuclein aggregates, between 1 and 12 months following α-synuclein fibril injections, and now report how far the pathology has spread 18- and 23-month post-injection in this model. Our data show that between 12 and 18 months, there is a further increase in the number of brain regions exhibiting pathology after human, and to a lesser extent mouse, α-synuclein fibril injections. At both 18 and 23 months after injection of mouse and human α-synuclein fibrils, we observed a reduction in the density of α-synuclein aggregates in some brain regions compared to others at 12 months. At 23 months, no additional brain regions exhibited α-synuclein aggregates compared to earlier time points. In addition, we also demonstrate that the induced α-synucleinopathy triggered a significant early neuron loss in the anterior olfactory nucleus. By contrast, there was no loss of mitral neurons in the olfactory bulb, even at 18 month post-injection. We speculate that the lack of continued progression of α-synuclein pathology is due to compromise of the neural circuitry, consequential to neuron loss and possibly to the activation of proteolytic mechanisms in resilient neurons of wild-type mice that counterbalances the spread and seeding by degrading pathogenic α-synuclein.

**Electronic supplementary material:**

The online version of this article (10.1007/s00401-017-1792-9) contains supplementary material, which is available to authorized users.

## Introduction

Parkinson’s disease (PD) is characterized by the accumulation of intraneuronal inclusions of alpha-synuclein (α-syn) in the somata (Lewy bodies) or in neurites (Lewy neurites) of neurons [18]. α-Syn, as a presynaptic protein, is natively unfolded, but in Lewy pathology, α-syn misfolds into amyloid fibrils that form the hallmark inclusions of PD [11, 14]. Post-mortem studies of people with PD suggest that α-syn pathology progresses in the brain in a stereotypical pattern over decades, first starting in the olfactory bulb (OB) and the dorsal motor nucleus of the vagus, and then spreading to central brain regions, ultimately reaching cortical areas [6–8, 13]. Observations also suggest that the propagation is occurring along neural connections, since the regions that are sequentially involved are also anatomically interconnected [13].

Severe α-syn pathology appears several years prior to the diagnosis of the classical motor symptoms of PD, and is accompanied by non-motor deficits such as olfactory dysfunction, constipation, and sleep disturbances [52]. One of the hallmarks of PD is a severe neuronal loss in the substantia nigra (SN) that leads to the loss of dopaminergic signalling and the appearance of motor symptoms. Neuronal loss is also observed in other brain regions in PD, such as the anterior olfactory nucleus (AON) [50], the locus coeruleus (LC) [17], and the amygdala [22]. In addition, the volumes of the piriform cortex (PC), the amygdala, the OB, and the orbitofrontal cortex are reduced [33, 50, 67, 74] (reviewed in [60]). The neuropathology in all of these brain regions likely contributes to the non-motor symptoms of PD [24, 54]. Based on the ability of misfolded α-syn to aggregate and further recruit endogenous α-syn, the α-syn transmission hypothesis posits that misfolded α-syn can form proteopathic seeds that template normal α-syn to misfold, undergo intra-axonal transport to other brain regions, and propagate synucleinopathies through iterative repetition of this process, perpetuating a vicious cycle [16, 20, 31, 40, 46]. This process is suggested to underlie the spatio-temporal progression of α-syn pathology observed in PD brains. Several laboratories have developed models of pathology induction and spreading by injecting recombinant fibrillar α-syn, or brain extracts from PD patients, into the brain, muscles, peritoneal cavity, or circulatory system of α-syn-overexpressing or wild-type rodents [2, 12, 36–39, 41, 49, 51, 56, 59, 61, 63]. These injections induce α-syn aggregate pathology in neuronal populations proximal to the injection site, and in more distant regions with neuronal connections to the site of injection. We recently demonstrated that pathology induced by unilateral injection of human or mouse wild-type α-syn pre-formed fibrils (PFFs), designated huPFFs or mPFFs, respectively, into the OB of 3-month-old wild-type (WT) mice can propagate sequentially over multiple synaptic relays, reaching numerous ipsi- and contralateral brain regions after 12 months, including brain stem areas (e.g., a few aggregates in the SN, LC, and raphe nucleus (RN)). The spreading of α-syn aggregates was coupled to progressive deficits in olfaction [59]. Taken together, the model involving huPFFs and mPFFs injections into the OB of WT mice results in the spread of pathology and olfactory deficits that mimic prodromal PD within 1–12 month post-injection [59]. In the present study, we further define the consequences of these huPFFs and mPFFs injections into the OB and determine, first, the extent of α-syn pathology at 18 and 23 months after injection and second, if there is cell loss in the olfactory bulb and in the AON.

## Materials and methods

### Study design

The goal of this study was to establish if the injection of PFFs into the OB of mice leads to neurodegeneration and what the pattern of α-syn pathology is 18–23 month post-PFFs injection. All experiments were performed blinded. After surgery, animals were assigned a name independent of the experimental group they were part of. That name was used for assessing pathology location and scoring. For other subsequent analysis, a second individual assigned new names to stained slides, so the experimenter would conduct the analysis blinded to the identity of the mice. Further experimental detail, protocols, including animal care/handling and number of biological/technical replicates, are presented in the following sections and in the figure legends.

### Animals

We used 48 C57Bl/6J female mice which were 3 months old at the start of the study, purchased from Charles River Laboratories (Sweden) and the Jackson Laboratory (USA). The mice had constant access to food and water, and were housed five to six per cage under a 12-h light/12-h dark.

### Stereotactic injections

We prepared PFFs from recombinant full-length mouse α-syn and full-length human α-syn. Purification and assembly of the proteins were performed as described [73]. PFFs and soluble mouse α-syn were stored at − 80 °C. On the day of the surgery, PFFs were thawed, sonicated as described by us previously [59], and kept at room temperature during the surgical procedure. Recombinant mouse wild-type α-syn monomers (mMs) used for controls was kept on ice during the surgical procedures.

Forty-two wild-type mice received stereotactic injections of sterile phosphate buffered saline (PBS), mMs, mPFFs, or huPFFs into the OB unilaterally, following our previously published protocol [58, 59]. Six additional mice did not undergo any surgical procedures (non-injected group, age-matched).

Some of the mice described in this study, which were left to live 1–12 months after surgery, are part of an experiment that we have reported on previously [59]. Additional 42 mice that were not euthanized prior to compiling our previous report were aged to a maximum of 23 month post-injection. For our current analyses, we used the following mice that were not part of our previous publication [59]: 9 months, 18–19 months (called “18 months” in the rest of the article), and 22–23 months (called “23 months” in the rest of the article) post-injection delays. In addition, we re-examined mice from the 1-, 3-, 6-, and 12-month-old groups from our previous work [59], for additional analyses (see Online Resource 1 for clarification). Only groups with three animals or more were used for analyses.

### Preparation of the tissue

Mice were perfused transcardially with 0.9% saline followed by 4% PFA; brains were collected and post-fixed as described previously [58, 59]. We stored the brains at 4 °C until sectioning. The entire brain of each mouse was sectioned using a freezing microtome into 30 μm free-floating coronal sections, and stored in antifreeze solution at 4 °C until immunostaining.

Sections from human brain with tau pathology (Alzheimer’s disease; angular gyrus) and sections from human brain with TDP-43 pathology (FTLD-TDP, cingulate cortex) were obtained from the Center for Neurodegenerative Disease Research, University of Pennsylvania, Philadelphia. These post-mortem brains came from longitudinally followed human subjects who were assessed neuropathologically as described [71]. Prior to 6 μm-thick sectioning, the samples were fixed in 10% neutral-buffered formalin, and paraffin embedded, and stored at RT.

### Immunohistochemistry

Paraffin embedded tissue was deparaffinised in xylene and rehydrated. For 1D3 and AT8 detection, heat-induced antigen retrieval was performed (citrate buffer, pH 6) prior to staining mouse and human samples. We stained coronal free-floating sections (mouse tissue) and sections on slides (human brain samples) using primary antibodies against phosphorylated α-syn (Ser129) (pser129, rabbit, 1:10000, Abcam, Ab51253), hyperphosphorylated TDP-43 (pS409/410) (TAR5P-1D3, rat monoclonal, 1:200, Ascenion, Munich, Germany) [45], or hyperphosphorylated tau (pS202/T205) (AT8, mouse monoclonal, 1:10,000, Thermo Scientific). Secondary biotinylated anti-rabbit serum (goat, 1:500, Vector Laboratories, BA-1000), anti-rat (goat, 1:500, Vector Labs, BA-9400), or anti-mouse (goat, 1:500, Vector labs, BA-9200) were used. We used a standard peroxidase-based method with DAB to detect the antibody (Vectastain ABC kit and DAB kit, Vector Laboratories). Slides were dehydrated and coverslipped with Cytoseal 60 mounting medium (Thermo Scientific) and analysed by the conventional light microscopy on a NIKON Eclipse Ni-U microscope (Nikon); we captured images with a Retiga 2000R digital camera using NIS Elements AR 4.00.08 software (Nikon).

For analysis of pser129 pathology spreading in the brains of mice, we stained with the anti-pser129 serum a whole series of coronal sections (210 µm intervals between consecutive sections) from 3 to 5 animals per group (non-injected, PBS-, mMs-, mPFFs-, and huPFFs-injected groups). We assessed, in a blinded manner, the presence of pser129-positive inclusions by screening every section at 20× magnification using a NIKON Eclipse Ni-U microscope.

### Immunofluorescence staining

We stained coronal free-floating sections with primary antibodies against pser129 (pser129, rabbit, 1:5000; Abcam, Ab51253) and NeuN (mouse, 1:1000, Millipore, Mab377), and secondary antibodies AlexaFluor 488 goat anti-mouse (1:500, Jackson Immunoresearch Laboratories, 115-545-146) and AlexaFluor 594 goat anti-rabbit (1:500, Jackson Immunoresearch Laboratories, 111-585-144). The staining with thioflavin-S was performed in accordance with our previous work [59].

Stained slides were blind coded before analysis and were imaged with an inverted confocal laser microscope Nikon Eclipse Ti-E. Thioflavin-S staining was imaged by averaging 4× scans for each z step of the stack to remove noise using NIS Elements AR 4.00.08 software (Nikon). For all other fluorescence staining, we acquired images by a single scan per stack step. We post-treated the confocal stacks with a kernel-3 median filter to remove noise and generated all final images using NIS Elements AR 4.00.08 software (Nikon).

### Scoring of pser129 pathology and heat map

**Fig. 1:**
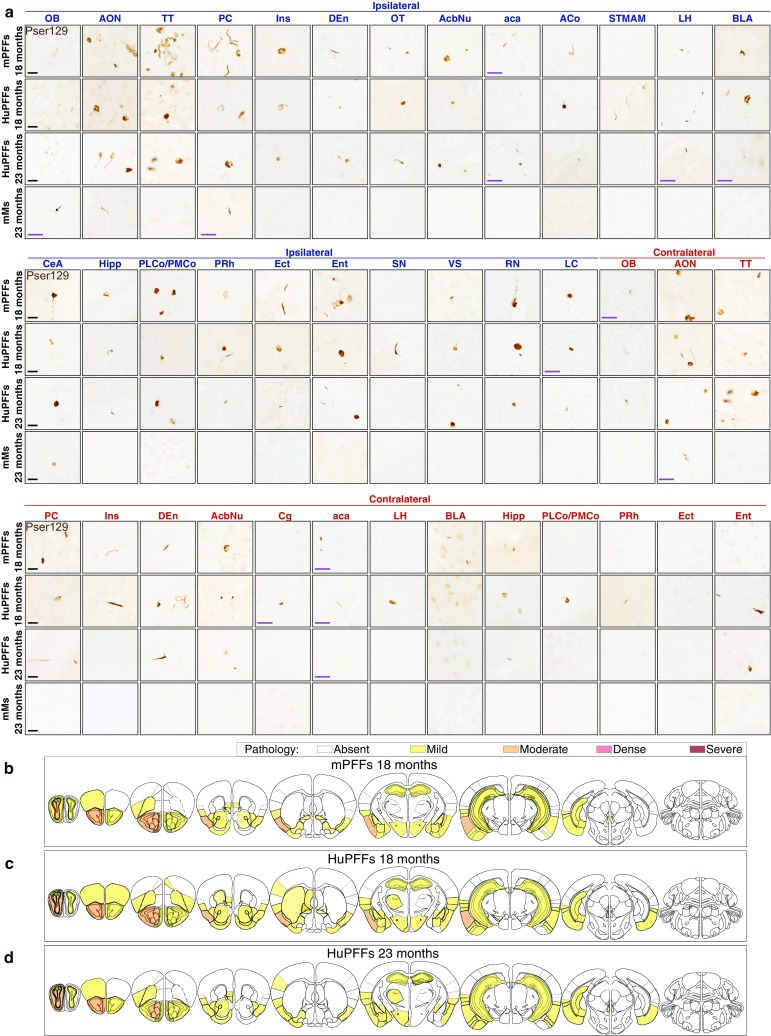
PFF-induced pathology at 18- and 23-month post-injection is mild (**a**) α-Syn pathology detected by an antibody against pser129 is present in brain regions ipsilateral (legend in blue) and contralateral (legend in red) to the injection site. Diagrams illustrating the anatomical location of α-syn pathology (assessed by antibody against pser129) in the brain on coronal sections at 18-month post-injection of mPFFs (**b**), of HuPFFs (**c**) and at 23-month post-injection of HuPFFs (**d**). The green star indicates the injection site. Severity of pathology is represented by different colours, from no pathology-to-severe pathology and is based on a scoring method. The sections were immunostained in three independent histochemical experiments. Histochemical analysis was performed on all animals at 18-month post-injection of mPFFs, and at 23-month post-injection of HuPFFs, mMs and PBS as well as in 21- and 26-month-old non-injected animals (age-matched; PBS-injected and non-injected mice not shown) (mPFFs 18 months: *n* = 3; HuPFFs 18 months: *n* = 5, HuPFFs 23 months: *n* = 4, mMs 18 months and 23 months: n = 4 for each time point; PBS 18 months: *n* = 3); non-injected mice 18-month post-injection time (age: 21 months): *n* = 3; 23-month post-injection time (age: 26 months): *n* = 3. The data presented here are from representative animals. Scale bar: 20 μm. All the images with a purple scale bar were acquired at ×60; all the other images were acquired at ×40 and match the black scale bars. A list of brain structure abbreviations is available in Online Resource 4

Neuropathology detected by antisera to pser129 was analysed on blind coded slides. We assessed the presence of pser129 inclusions on a whole series of coronal sections (210 µm intervals between sections) from 3 to 5 animals per experimental group, employing a simplified version of the method used previously [59]. We screened every single section at 20× magnification using a NIKON Eclipse Ni-U microscope and we assigned a score to each brain region, from 0 to 4, based on the relative abundance of pser129-positive inclusions (cell bodies and neurites) (0 = no aggregates, 1 = sparse, 2 = mild, 3 = dense, 4 = very dense). Brains regions in the diagrams presented on Fig. 1b–d were colour coded according to the score from 0 to 4 that represent the median of animals per group.

To generate a heat map (Fig. 3a–c), we calculated the average score values per group per brain region [27], and represented the data on a heat map programmed with the software R v3.2.1 [53] (https://cran.R-project.org/). Representative images of pser129 staining presented in Fig. 1a were acquired at 40× or 60× magnification on the same microscope.

### Densitometry of pser129 pathology measured by ImageJ

We assessed pser129 pathology density in the OB, the AON, the anterior and posterior PC and the entorhinal cortex (Ent) in mMs-, huPFFs-, mPFFs-injected mice 12- and 18-month post-injection, and in non-injected age-matched animals. 20× magnification images were acquired using a NIKON Eclipse Ni-U microscope (Nikon), using the same exposure time for all the images. OB images were acquired using the condenser, while images of the AON, PC, and Ent were acquired without the condenser lens (to take into account a thicker layer of the section). In the OB, images were acquired on three sections separated by 420 μm intervals (between Bregma 4.9 and 4 mm), the last section being the most posterior section before the appearance of the AOB (Bregma 4 mm approximately). Three images of the medio-lateral AON were acquired (420 µm intervals between sections, covering the whole AON; between Bregma 3 and 2 mm). For the Ent, three images were acquired (840 µm intervals between sections imaged, between Bregma − 2.90 and − 4.40 mm). We analysed separately the anterior and the posterior PC, since they receive inputs from different brain regions (for review, [5]). We acquired four pictures of the anterior PC (630 µm intervals between sections imaged, localized between Bregma 2.34 and 0.2 mm); and four pictures of the posterior PC (630 µm intervals between sections imaged, localized between Bregma 2.4 and 0.34 mm). Images were then processed in ImageJ64 [55] as described in Online Resource 2, to create a mask (to exclude the background) that redirects to the original image for analysis. For brain regions like the PC that might not fit entirely in the field, we drew the contour of the region, and the analysis was performed within the contoured area (Online Resource 2b), we measured the area and the mean grey value of the area that was positively stained. We then calculated the grey value per square pixels per image (A.U./px^2^ = mean grey value × area stained/total area assessed). We then calculated the average grey value per square pixels for each brain region of each animal (*n* = 3–5/group, details in the legend of Fig. 4). We then calculated the average grey value per square pixels for each group and region of interest. Individual data were then analysed using linear mixed-effects models with a random intercept for each sample via the ‘lme4′ (http://lme4.r-forge.r-project.org/) package in R. Linear contrasts with false discovery rate adjustments were used to test specific hypotheses and account for multiple testing (brain region, time point, experimental group, and ipsi- versus contralateral region). The inclusion of the random intercepts accounts for the auto-correlation in these data and ultimately yields better model performance and increased accuracy [30]. Normality and homoscedasticity assumptions were verified using quantile–quantile plots and scattered plots of the residuals. Outcomes were natural log transformed, which improved modelling assumptions. For ease of interpretation, effects were back-transformed to estimate fold change and the 95% confidence interval. Graphs were prepared using Prism 6.0, GraphPad.

### Mitral cell density

We measured the density of mitral cells in the OB on cresyl violet-stained sections. The staining and the analysis were performed as described previously [59]. Homoscedasticity was verified with a Brown–Forsythe test. We then performed a two-way ANOVA with repeated measures using Prism 6.0, GraphPad. As no significant effect of PFFs injection was observed, we did not perform any post hoc tests.

### Cresyl violet-positive cell counting by stereology in the AON

Cresyl violet stains all cells including large neurons, small neurons, and glial cells that cannot be distinguished by morphology from the small neurons (Fig. 5a, arrows) [29]. The AON contains small-sized interneurons [3] (subpopulation of GABAergic neurons [28]), which are similarly sized to glial cells. The glial population has been estimated to account for 10–36% of the total cell population in the mouse brain [23, 44]. We quantified cresyl violet-positive cells by stereology in the ipsilateral and contralateral AONs of mMs-, mPFFs-, huPFFs-injected mice 6 and 18 month post-injection, and non-injected age-matched controls. Large neurons with faint cresyl violet-positive signal (indicated by arrowheads on micrographs in Fig. 5a) and small dark stained cells (indicated by arrows on micrographs in Fig. 5a) were quantified separately. Hence, our quantifications of the dark stained cells likely also include glial cells that account for only a small proportion of cells present in the AON, but the large faint cresyl violet-positive cells include only neurons. We used a computer-assisted mapping and cell quantification program (Stereo Investigator, MBF Bioscience, Williston, USA) coupled to a Zeiss Imager M2 microscope (Zeiss, Thornwood, NY). We analysed a full series of sections (5–7 sections) per animal spaced by 210 μm (section interval = 8), in 3–5 animals per group. Contours of the region were drawn at 2.5× magnification. Quantifications were performed at 100× (oil objective) using a counting frame of 40 μm × 40 μm, grid size was set to 100 (medial–lateral) × 140 (dorsal–ventral) μm, with a guard zone of 3 μm, and dissector height set at 8 μm. These parameters used for AON stereology analyses were set to assess at least 300 total cells per animal and side, and have an error coefficient (*m* = 1) below 0.09. We then calculated the average cell number per group and region of interest and analysed the data using negative binomial mixed-effects models with random intercepts for each individual, using the ‘lme4 package (http://lme4.r-forge.r-project.org/) in R v3.2.1 (https://cran.r-project.org/). A negative binomial regression was chosen, because these are count data and the mixed effects allow higher performance of the model and increased accuracy [30]. Linear contrasts with false discovery rate corrections were used to adjust for multiple testing (cell morphology, time point, experimental group, and ipsi- versus contralateral AON). Normality and homoscedasticity assumptions were verified using quantile–quantile plots and scatter plots of the residuals. Graphs were prepared using Prism 6.0, GraphPad.

## Results

We hypothesised that α-synucleinopathy would continue to spread at survival times exceeding 1 year post-injection, i.e., beyond the brain regions previously reported to be affected 12 months after such injections [59]. We previously reported that after 3 months following mPFFs injection, severe and widespread α-syn pathology in many regions directly or indirectly connected to the injection site was observed [59]. After 12 months, α-syn pathology induced by mPFFs reached more than 40 different brain subregions, including contralateral structures [59]. In the current study, we also hypothesise that following injection of either huPFFs or mPFFs α-syn into the OB of wild-type mice, α-syn aggregation in olfactory pathways would lead to neuron loss, which could contribute to the olfactory deficits previously described [59]. In our earlier study, we demonstrated that there is no loss of mitral cells in the OB at 6 month post-injection [59].

### Absence of pser129-unspecific staining in white matter tracts

As in our previous work [59], we detected pathology using an antibody against pser129 α-syn. This post-translational modification of α-syn is indicative of abnormal α-syn in synucleinopathies [15, 48] and is a reliable marker for α-syn inclusions [4, 57]. Concerns have been raised previously about pser129 antibodies and their possible binding to phosphorylated neurofilament subunit L in white matter tracts [64]. Therefore, we tested our pser129 staining protocol in white matter and found that it results in no detectable staining in different white matter tracts of non-injected animals and mMs-injected animals (Online Resource 3a).

### Widespread mild α-syn pathology is present at 18 and 23 month post-injection

When assessing pathology in the whole brain of PFFs-injected mice, we observed widespread pser129-positive pathology in neurites and cell bodies including ipsilateral and contralateral olfactory regions, and non-olfactory regions (Fig. 1a). We analysed at least one series of sections from the entire brain of all groups of mice, i.e., PBS-, mMs-, huPFFs-, and mPFFs-injected and non-injected age-matched mice. Consistent with the strategy used to assess α-syn pathology in publications on human synucleinopathy brains [4, 6, 8], and our own prior work [59], we considered the presence of a single pser129-positive cell or neurite to indicate that pathology was present.

α-Syn pathology was present in numerous brain regions 18 months after mPFFs and huPFFs injection and 23 months after huPFFs injections (Fig. 1a), including in olfactory regions and non-olfactory regions that are directly and indirectly or transneuronally connected to the injected OB. We scored the severity of α-syn pathology in the entire mouse brain [59]. At 18- and 23-month post-injection, the α-syn pathology in mice injected with PFFs was mild-to-moderate (Fig. 1b–d).

In addition, we assessed several control groups (mMs, PBS-injected mice, and non-injected age-matched mice). PBS-injected mice and non-injected mice did not display signs of α-syn pathology at 18- and 23-month post-injection time points (data not shown, *n* = 3–4 per group). Two out of four mice injected with mMs assessed at 18-month post-injection, and one mouse out of four assessed at 23-month post-mMs-injection exhibited a very low number of pser129-positive neurites in the ipsilateral OB, PC, and central amygdala (CeL) (Fig. 1a).

### Pser129 inclusions at 18 months are present within neurons, and are positive for thioflavin-S

**Fig. 2:**
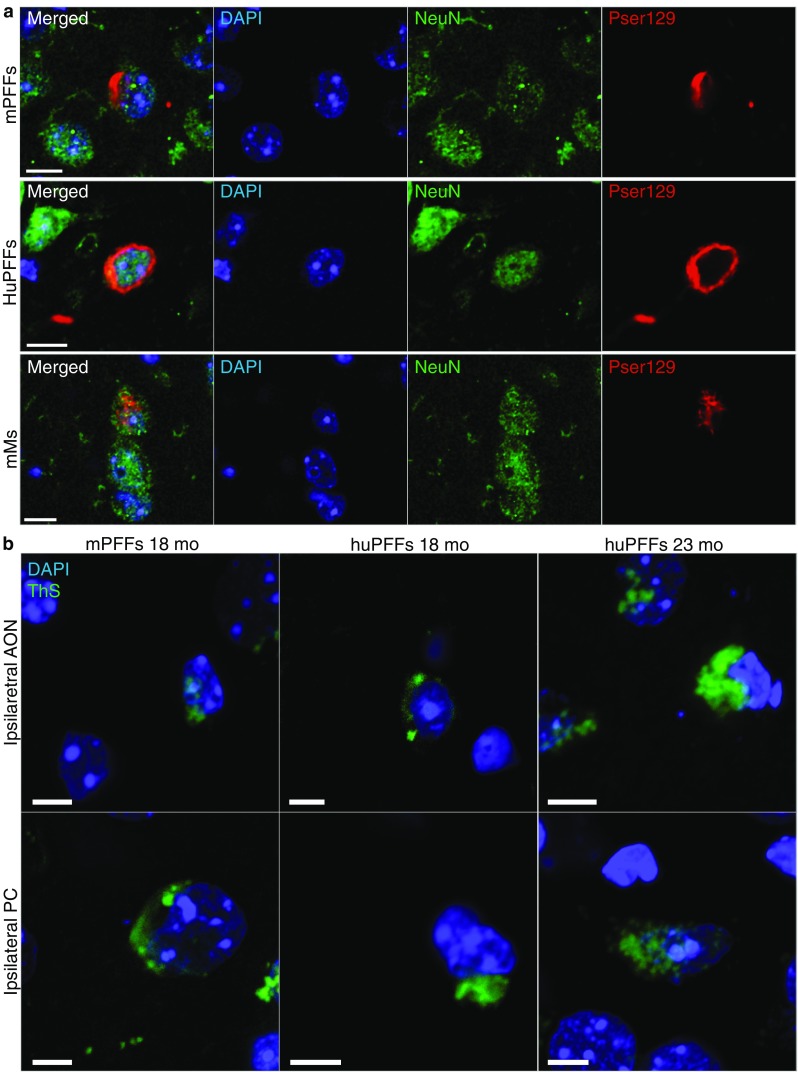
Pser129-positive inclusions induced by injected PFFs or mMs are localized within neurons, and are thioflavin-S-positive. **a** Pser129-positive inclusions (red), NeuN-positive cells (green), and nuclei stained by DAPI (blue) in the ipsilateral AON 18 months after injection of mPFFs, HuPFFs, or mMs. The sections were immunostained in two independent histochemical experiments. Histochemical analysis was performed on 3 animals per group 18-month post-injection of mPFFs, HuPFFs, and mMs. **b** Images from confocal stacks showing examples of thioflavin-S-positive inclusions detected in the ipsilateral AON and PC of PFFs-injected mice (Thioflavin-S, green; DAPI-positive nuclei, blue). Three animals per group (mPFFs 18 months, huPFFs 18, and 23 months) were stained and analysed in a single experiment. Scale bars: **a** 10 μm; **b** 5 μm

Next, we used confocal microscopy to determine whether pser129-positive inclusions are present within the neurons of the AON. As expected, and consistent with our previous report [59], NeuN-positive cells in the AON displayed pser129-positive inclusions (Fig. 2a). We also observed pser129-positive inclusions in non-NeuN-positive cells (data not shown).

To confirm that the inclusions observed with pser129 antibody in our model are mature amyloid inclusions, we performed a staining with thioflavin-S (Fig. 2b). Inclusions in PFFs-injected mice 18- and 23-month post-injection are thioflavin-S-positive, indicating mature amyloid structures.

### PFF-induced pathology does not trigger the aggregation of tau and TDP-43

Since TDP-43 and tau pathology co-occur with α-syn pathology in PD, PDD, and DLB in humans [43, 69], and the presence of tau pathology influences α-syn pathology load in PDD [26, 70], we investigated whether PFFs injections could trigger tau and TDP-43 pathology after long post-injection times. We assessed tau and TDP-43 pathology in mice injected with PBS, huPFFs, and mPFFs 18-month post-injection. Tau and TDP-43 pathologies were detected by immunohistochemistry against hyperphosphorylated tau (AT8), and against hyperphosphorylated TDP-43 (1D3), respectively, as commonly used [19, 45]. Sections from post-mortem tissue of a person with Alzheimer’s disease (AD) with abundant neurofibrillary tangles and of a person with frontotemporal lobar degeneration (FTLD) with severe TDP-43 pathology were used as positive controls for the staining procedure. While tau and TDP-43 pathology were evident in human tissue, no pathology could be detected in PFFs-injected mice (Online Resource 3b), indicating that PFFs injections did not trigger tau and TDP-43 pathology 18-month post-injection.

### Changes in α-syn pathology across time

**Fig. 3:**
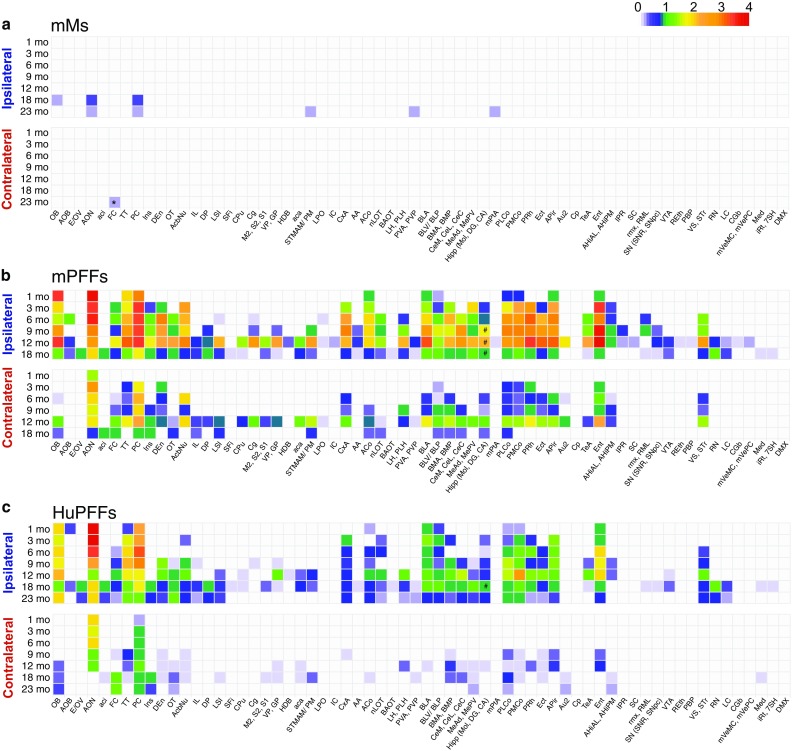
PFF-induced pathology propagates 18 months after injection. Heatmaps showing the severity of pathology (on a scale from 0 to 4; data shown as the mean score of each group) in various brain regions ipsilateral (legend in blue) and contralateral (legend in red) to the site of injection, from 1- to 23-month post-injection of mMs (**a**), from 1- to 18-month post-mPFFs injection (**b**) and from 1- to 23-month post-injection of HuPFFs (**c**). The scoring was performed on all animals at 9 and 18-month post-injection of mPFFs, and at 23-month post-injection of HuPFFs, mMs, and PBS as well as in 12-, 21-, and 26-month-old non-injected animals (age-matched controls; PBS-injected and non-injected mice not shown) [mPFFs 9 months: *n* = 5, HuPFFs 9 months: *n* = 5, mMs 9 months: = 5, PBS 9 months: *n* = 4; mPFFs 18 months: *n* = 3; HuPFFs 18 months: *n* = 5, HuPFFs 23 months: *n* = 4, mMs 18 months and 23 months: *n* = 4 for each group; PBS 18 months: *n* = 3, non-injected mice 21-month-old (age-matched to 18-month post-injection groups): *n* = 3; non-injected mice 26-month-old (age-matched to 23-month post-injection groups): *n* = 3]. For 1, 3, 6, and 12 months delays, the average scores were calculated from analysis on animals presented in our previous article [delay 1, 3, 6, 12 months, [59] (*n* = 4–5 animals per group). All the animals (1- to 23-month delays) are part of the same experiment (divided in four different experimental sessions). * Mild pathology is observed on contralateral frontal cortex in only 1 mM-injected mouse out of 4, at 23-month post-injection. # At extended time points, the ipsilateral hippocampus (Hipp) exhibits pathology in the radial, molecular, pyramidal, oriens’ layers, the cornu ammonis, dentate gyrus, and the pyramidal and molecular layer of the dentate gyrus. A list of brain structure abbreviations is available in Online Resource 4

To investigate the kinetics of α-syn pathology propagation after long delays post-injection, we calculated the mean score of pathology (scoring in all the brain regions where pathology could be detected at any time between 1- and 23-month post-injection. Data are summarised in heatmaps (Fig. 3) and include 18- and 23-month-old animals presented in this study, animals assessed at 1, 3, 6, and 12 months old that were described in our previous article [59], and an additional time point, at 9-month post-injection. Heatmaps are based on the same scoring method as the diagrams (Fig. 1b–d) but provide a better overview of the data.

No pathology was detectable at 1-, 3-, 6-, and 12-month post-injection in mM-injected mice (score 0) (Fig. 1a, [59]). As expected, no pathology was detected at 9-month post-injection (Fig. 1a), but mild pathology (mainly in neurites, occasionally in the cell body) appeared in olfactory regions in mice injected with mMs at 18-month post-injection, and in additional non-olfactory regions at 23 months (Fig. 3a) (score between 0 and 1).

#### mPFFs injection

Mice injected with mPFFs progressively developed severe and widespread pathology between 1- and 12-month post-injection (Fig. 3b, [59]), exhibiting pathology scores between 2 and 4 in many brain regions after 6-month delay. At 18-month post-mPFFs injection, we observed α-syn pathology in brain regions that did not contain α-syn pathology at 12 months (Figs. 1a, b, 3b, [59]) (i.e., ipsilateral peduncular cortex (DP), retromammilary nucleus (RML), ipsilateral and contralateral infra-limbic cortex (IL), contralateral OB, and contralateral anterior part of the paraventricular thalamic nucleus (PVA), indicating a progressive spread of α-syn pathology. By contrast, some regions that exhibited α-syn pathology at 12-month post-injection (Fig. 3b, [59]) did not display pser129 inclusions at 18-month post-injection, particularly some that are indirectly connected to the injection site (Fig. 3b) (e.g., ipsilateral thalamus (Th); association area of the temporal cortex, TeA; SN; LC; contralateral entorhinal, Ent, and ectorhinal, Ect, cortices). An apparent decrease in the pathology scores after 18 months suggests a reduction in the density of pathology in specific brain regions compared to the 12 months delay.

#### HuPFFs injection

Next, we assessed α-syn pathology following the injection of huPFFs into the OB. Between 1- and 12-month post-injection, the pathology score remained mild (between 0 and 2) in most of the brain regions affected, except the ipsilateral AON and the PC, with scores up to 4 (Fig. 3c). α-Syn pathology after 18 months emerged in many previously spared brain regions (e.g., ipsilateral and contralateral insular cortex (Ins), ipsilateral primary and secondary sensory and motor cortical areas (S1, S2, M1, M2, respectively), SN, RN, LC, iDP, IL, RML; and contralateral central nuclei of the amygdala (CeL, CeM, CeA), Hipp (molecular layer, dentate gyrus and Cornu Ammonis 2), lateral hypothalamic area (LH), and peduncular part of the lateral hypothalamic area (PLH) and olfactory tubercle (OT) (Fig. 3c). At 23-month post-injection, pathology was still present in numerous brain regions, but no longer observed in several previously affected brain regions (e.g. ipsilateral M1; M2; S1; S2; TeA; SN; caudate putamen, CPu; anterior and posterior basomedial nuclei of the amygdala, BMA, BMP; contralateral Ins; anterior, ventral and posterior basolateral amygdala, BLA, BLV, BLP) (Fig. 3d), which may indicate a regression of pathology at extended periods after PFFs injection. In analogy to our findings after mPFFs injections, huPFFs-injected mice displayed mild-to-moderately severe pathology at the 18- and 23-month time points (Fig. 3b, c). The present results suggest that the amount of α-syn pathology induced by huPFFs in a given brain region does not increase further beyond what is seen 1 year after injection, despite a greater number of brain regions being affected at 18 months.

### α-Syn pathology density

**Fig. 4:**
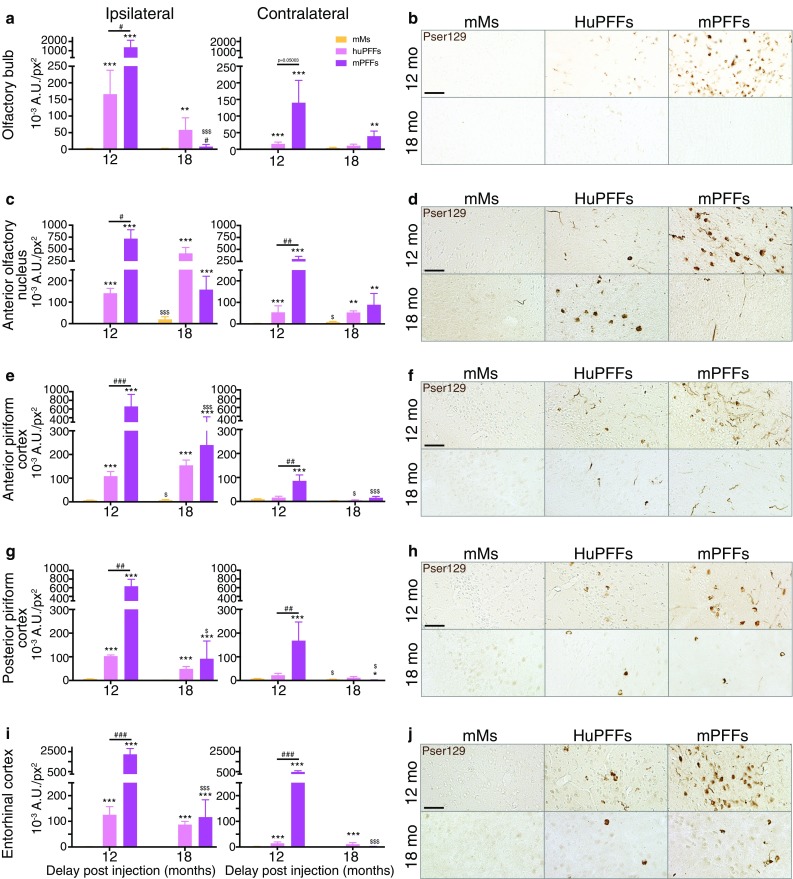
Pathology load decreases at 18 months post-injection in specific brain regions. Densitometry of α-syn pathology (pser129 staining) measured in ipsi- and contralateral OB (**a**), AON) (**c**), anterior PC (aPC) (**e**), posterior PC (pPC) (**g**), and Ent (**i**) at 12- and 18-month post-injection of mMs, HuPFFs, and mPFFs. The sections were immunostained in seven independent histochemical experiments. Representative images of the average densitometry of pathology in the OB (**b**), AON (**d**), the aPC (**f**), pPC (**h**), and Ent (**j**). Densitometry of pser129 pathology in the OB, AON, aPC and pPC, and Ent was measured in all animals at 12- and 18-months post-injection of mMs, HuPFFs, or mPFFs (mPFFs 12 months: *n* = 4; mPFFs 18 months: *n* = 3; HuPFFs 12 months: *n* = 5; HuPFFs 18 months: *n* = 5; mMs 12 months: *n* = 5; mMs 18 months: *n* = 4). For the PC, the anterior PC and the posterior PC were examined separately, and no significant difference between them was observed. Statistical analyses were performed by linear mixed-effect models (regression and contrasts) to include three factors: delay post-injection, ipsi/contralateral, and injectate. Statistical data are available in Online Resource 5. * *p* < 0.05, ** *p* < 0.01, *** *p* < 0.001 in comparison to mMs-injected mice at a given time point and side of the brain. ^#^ *p* < 0.05, ^##^ *p* < 0.01, ^###^ *p* < 0.001 for comparisons between mPFFs- and huPFFs-injected mice at a given time point and side of the brain. ^$^ *p* < 0.05, ^$$^ *p* < 0.01, ^$$$^ *p* < 0.001 for comparison between 12- and 18-month delays for a given mouse group and side of the brain. Scale bar: 50 μm

To further investigate the changes in α-syn pathology across time and the potency of the different PFFs to induce pathology, we measured the density of pser129-positive inclusions 12- and 18-month post-mMs, huPFFs, and mPFFs injection in the OB, the AON, the PC, and the Ent (Fig. 4) (methods summarised in Online Resource 2, statistical analyses are detailed in Online Resource 5).

First, as expected, the density of pathology was significantly higher in the ipsilateral side than the contralateral side after injection of huPFFs and mPFFs (Fig. 4, Online resource 5a). In the mMs-injected group, pathology is very sparse or absent. HuPFFs induced significantly more pathology than mMs, while mPFFs induced up to ten times more abundant pathology than elicited by huPFFs at 12-month post-injection (Fig. 4, Online resource 5b), indicating that mPFFs were more potent than huPFFs.

The pathology induced by mMs increases significantly between 12 and 18 months in the AON and the PC, while pser129 burden did not change between 12 and 18 months after huPFF injection (Fig. 4, Online resource 5c). In contrast, mPFFs-induced pathology decreased significantly at 18 months in the ipsilateral OB (Fig. 4a, b, Online resource 5c), in the ipsilateral and contralateral anterior PC (Fig. 4e, f, Online resource 5c), posterior PC (Fig. 4g, h, Online resource 5c) and Ent (Fig. 4i, j, Online resource 5c), reaching similar levels as huPFFs (AON, aPC, pPC, and Ent).

We also compared the density of pathology between the anterior and posterior PC, and no differences were observed (Online resource 5d). Altogether, our results confirm the decrease of pathology density after 18-month post-mPFFs injections, and demonstrate that there is no further increase in the density of α-syn pathology induced by huPFFs injection past 12-month post-injection, despite the involvement of additional brain regions (Fig. 3).

### Severe cell loss in the AON but not in the OB

To explore if neural cell death contributes to the olfaction deficits which we previously reported [59], we assessed cell loss by stereology at 6- and 18-month post-injection in the OB and in the AON. The AON receives direct projections from the OB and coordinates the activity of the olfactory system between both hemispheres [9].

**Fig. 5:**
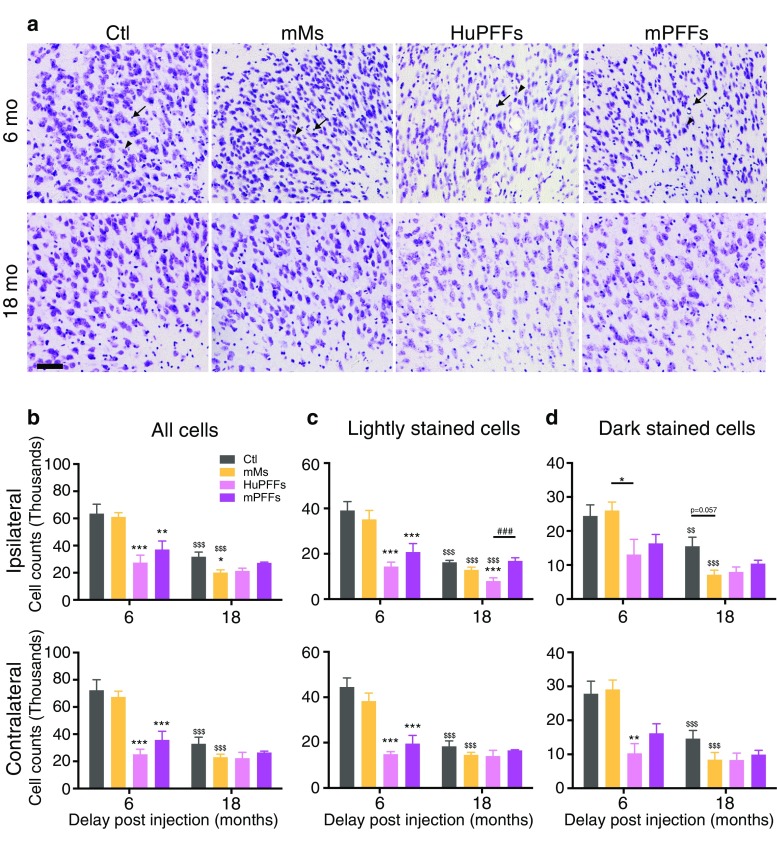
Cell loss in the AON measured by stereology. **a** Representative images of cresyl violet staining in the AON of mMs-, HuPFFs-, and mPFFs-injected mice, 6- and 18-month post-injection and in age-matched non-injected mice (Ctl; 9 and 21 months old). **b**–**d** Cresyl violet-positive cell counts assessed by stereology in the ipsilateral and contralateral AON of these mice. **b** Total number of cresyl violet-positive cells. **c** Subpopulation of large cells (identified by arrowheads in **a**). **d** Subpopulation of small dark cells (identified by arrows in **a**). Data were analysed by negative binomial mixed-effects model to include three factors: delay post-injection, ipsi/contralateral side, and injectate. **a**–**d** Sections were stained in five independent histological experiments. Stereological analysis was performed on all animals at 6- and 18-month post-injection of mPFFs, HuPFFs, and mMs (6-month post-injection: mMs: *n* = 5, mPFFs: *n* = 5, HuPFFs: *n* = 4, 18-month post-injection: mMs: *n* = 4, mPFFs: *n* = 3, huPFFs: *n* = 5), and in age-matched non-injected mice (*n* = 3 per age). Statistical data are available in Online Resource 6. * *p* < 0.05, ** *p* < 0.01, ***p < 0.001 in comparison to mMs-injected mice and to non-injected mice at a given time point and a given side of the brain. ^#^ *p* < 0.05, ^##^ *p* < 0.01, ^###^ *p* < 0.001 for comparisons between mPFFs- and huPFFs-injected mice at a given time point and side of the brain. ^$^ *p* < 0.05, ^$$^ *p* < 0.01, ^$$$^ *p* < 0.001 for comparison between 12- and 18-months delays for a given mouse group and a given side of the brain. Scale bar: 50 μm

In the ipsilateral AON, the total number of cresyl-positive cells 6-month post-injection decreased by 55–58% after huPFFs injection and by 39–42% after mPFFs injection (Fig. 5a, b, Online resource 6b). In the contralateral AON, the number of cresyl violet-stained cell 6-month post-injection decreased by 61–62% in huPFFs-injected mice and by 47–51% in mPFFs-injected mice (Fig. 5a, b, Online resource 6b). However, there were no significant differences between huPFFs and mPFFs in the ipsi- and contralateral AON (Online resource 6b). Differences between ipsilateral and contralateral AON were not significant either (Online resource 6a), indicating that despite unilateral injection, both sides were affected similarly and severely at 6-month post-injection. At 18-month post-injection, huPFFs and mPFFs groups showed no further decrease in cell loss compared to 6 months (Fig. 5b, Online resource 6c), suggesting that severe PFF-induced cell loss occurred within 6 months after the injection only. Surprisingly, we observed a 50–77% decrease in total cell number between 6 and 18 months in the control groups (mMs-injected and non-injected aged match controls) indicating cell loss in the AON due to aging (Fig. 5b, Online resource 6c).

Since cresyl violet labels both neuronal and non-neuronal populations, we also quantified cells by their appearance: lightly stained cells (neuronal population, Fig. 5c) and dark stained cells (which include glial cells, and small interneurons; Fig. 4d) (for details, see “Materials and methods”). Both populations of lightly stained cells and dark stained cells are affected by age and by the injectate; indicating that pathology induced by PFFs injections does not affect a specific cell population defined by its morphology.

**Fig. 6:**
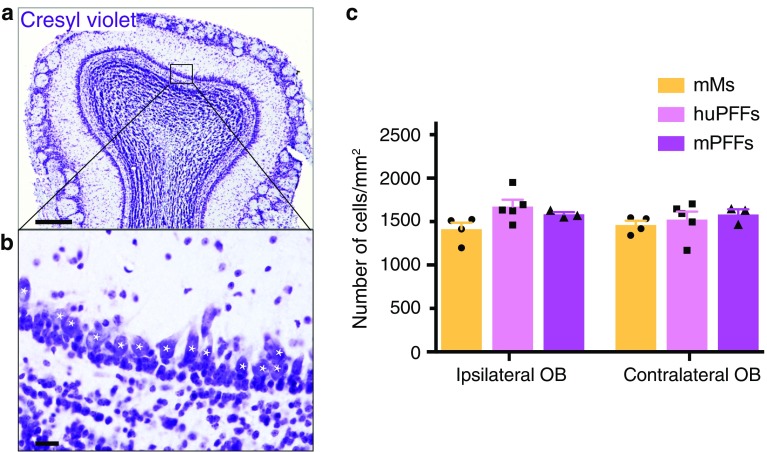
No loss of mitral cells in the OB at 18-months post-injection of PFFs. **a**, **b** Cresyl violet staining in the OB from one representative animal at low magnification (**a**), and high magnification (**b**) showing mitral cells (cell bodies identified by white stars). **c** Density of mitral cells in the mitral layer of the OB 18-month post-injection of HuPFFs, mPFFs, and mMs. We found no significant evidence of heteroscedasticity using a Brown-Forsythe test. We then performed two-way ANOVA with repeated measures where no significant effect was observed. (Group effect: *F*(2.9) = 1.840 *p* = 0.2138, ipsi/contralateral effect: *F*(1.9) = 0.6184 *p* = 0.4519, Interaction: *F*(2.9) = 2.082 *p* = 0.1807). The sections were stained in two independent histological experiments. Stereological analysis was performed on all animals 18-month post-injection of mPFFs, HuPFFs, and mMs. (mMs: *n* = 4, mPFFs: *n* = 3, HuPFFs: *n* = 5). Scale bars: **a** 500 μm; **b** 20 μm

We then investigated the mitral cells of the OB, which are the OB relay neurons that project to secondary olfactory regions, and are easily identified by their characteristic morphology (Fig. 6a, b). We previously reported no loss of mitral cells 6-month post-injection [59]. Those findings indicated that the injection process itself and the α-syn pathology that was evident in the OB 1–6-month post-injection were not deleterious to mitral cells. To confirm that mitral cell loss in the OB is not simply delayed compared to cell loss that we measured in the AON, we quantified the density of cresyl violet-positive mitral cells in the OB at 18-month post-injection (Fig. 6). Again, we observed no significant differences in mitral cell density between the groups at 18 months, and the densities were in the same range (Fig. 6c) as those we previously reported for mice at 6-month post-injection [59]. This confirms that it is unlikely that mitral cell loss in the OB contributes to the behavioral deficits which we have previously described in this model [59].

## Discussion

We performed carefully controlled microinjections of huPFFs and mPFFs assembled from recombinant α-syn monomers [59] to trigger α-synucleinopathy in the OB of wild-type mice. Building on our previous report where we observed progressive worsening of α-synucleinopathy and olfactory deficits over 1 year after injection, we now observe mild propagation following longer delays. At 18 and 23 months after injection, in general, α-syn inclusions appeared in additional brain regions compared to the results at 12 months, but within several brain regions previously affected, the density of the α-syn pathology decreased. We also observed marked cell loss in the AON at 6 and 18 months.

### Pser129 inclusions at extended time points post-injection are mature inclusions

As expected, the pser129 inclusions present 18- and 23-month post-injection were located in neurons and detectable by an antibody against α-syn and thioflavin-S staining, confirming that they were mature. These inclusions had characteristics similar to those of inclusions 1-month post-PFFs injection [59]. As mentioned earlier, in the AON, we observed additional inclusions that were not co-localized with the NeuN marker. Since neuronal populations localized in the AON are all labelled by the marker NeuN [42, 66, 75], our observations suggest that non-neuronal cells can also contain α-syn inclusions at 18 months. This is consistent with the observation of inclusions in oligodendrocytes and astrocytes in synucleinopathies and with the previous reports from cell culture and animal models (reviewed in [10]). An alternative explanation could be that these inclusions are localized in microglia that can phagocytose aggregates in PD [65]. Another possible explanation is that the cells bearing the inclusions are dying neurons that stopped expressing the NeuN marker [21, 32].

### Increased number of brain regions is affected by α-synucleinopathy after 18 months but not after 23 months

We then assessed α-syn pathology 18- and 23-month post-injection of PFFs. Compared to mPFFs-induced pathology spreading observed between 1- and 12-month post-injection (Fig. 3, [59]), five additional brain regions display pathology at 18 months (i.e., ipsilateral DP, RML, ipsilateral and contralateral IL, and contralateral OB and Th). Among these regions, some have direct connections with the ipsilateral OB or with other olfactory regions (DP, IL; [72]), and thus could be expected to develop pathology at an earlier time point. Notably, the contralateral OB developed pathology only at 18 months, although it had received direct projections from the contralateral AON, which had already developed pathology at 1-month post-injection, and it receives feedback projections from other contralateral olfactory regions that developed pathology 3-month post-injection [59]. By contrast, some regions that contained α-syn pathology at 12-month post-injection of mPFFs [59] did not exhibit pser129 inclusions at 18-month post-injection (e.g., ipsilateral Th; TeA; SN; LC; contralateral Ent and Ect).

Compared to mPFFs, we previously showed that huPFFs induced less widespread pathology after 12 months [59]. Here, we demonstrate that huPFFs-induced pathology progresses further at 18 months, appearing in many additional brain regions including the ipsilateral Ins, S1, S2, M1, M2, LC, SN, RN, and contralateral OT, dentate gyrus, Cornus Ammonis 2, molecular layer of the Hipp, and LH, thus reaching a spatial extent comparable to what was observed 12 months after mPFFs injections [59]. However, at 23-month post-huPFFs-injection, pathology does not progress further anatomically, and several brain regions that contained pathology at 18 months no longer display pathology (e.g., sensory and motor cortices, SN, basomedio- and lateral nuclei of the amygdala, and CPu), suggesting a regression of the spatial extent of pathology. Brain regions that showed only transient pathology at 12-month post-mPFFs injection and 18-month post-huPFFs injection contained very mild pathology. These observations suggest that, in this model, particular brain regions that exhibited a small amount of pathology after injection may be able to clear pathological inclusions and stop the development of additional inclusions. The precise mechanism for the clearance of inclusions in our model is unknown, but we know from other studies that the cells themselves are able to degrade (parts of) inclusions via autophagy–lysosomal and ubiquitin–proteasome degradation systems [76]. This could contribute to a decrease of inclusions in specific brain regions in our model. In addition, cells containing inclusions might degenerate, as described previously in another PFFs-model [47]. Finally, microglial cells are able to clear dead cells and debris (for review, see [10, 77] and astrocytes were shown to degrade α-syn aggregates in cell culture systems [35], suggesting that both of these cells types might contribute to clearing inclusions in our model. Finally, the effect of PFFs injection on the expression of endogenous α-syn in WT mice has not yet been investigated. It is conceivable that PFF-related pathology could suppress the expression of endogenous α-syn in our model and reduce the availability of endogenous α-syn to form aggregates. Supporting this idea, the work by Lim and colleagues [34] demonstrated that the suppression of α-syn expression for 3 months in an α-syn inducible transgenic mouse model leads to a decrease in α-syn pathology in the brain and reverses synaptic defects and memory deficits.

In our study, while PBS-injected or non-injected groups did not exhibit pser129 inclusions, some mice that received injections of mMs (18-month post-injection: 2 mice out of 4; 23-month post-injection: 1 mouse out of 4) showed rare pser129-positive inclusions in some olfactory regions (see Fig. 1a). Previously, we studied mMs-injected mice from 1 to 12 months, and did not observe pathology within this time range [59]. Others have reported the development of pser129-positive inclusions in cortical areas and the SN at 6 months after injection of non-fibrillized α-syn into the striatum of rats [49] or that soluble α-syn injected into transgenic mice overexpressing α-syn led to α-syn aggregation at 4-month post-injection [62]. It is possible that aggregation of mMs may have occurred in the test tube just prior to the injection or after injection, given the high concentration of monomers present in solution. Differences in the host (rats or transgenic mice in the other studies, versus wild-type mice in our studies) might explain why in our experiment mMs induced occasional pathology so late after the injection (18 months) compared to the previous literature (4–6 months).

Overall, our observations in this study and in our previous work [59] strongly support a propagation of pathology between brain regions connected via neuronal pathways. However, we cannot rule out that other mechanisms might be involved. An alternative explanation is the idea that all the brain areas that eventually develop aggregates are exposed to PFFs immediately following PFFs injection, but develop pathology in a sequential manner. This hypothesis does not explain why pathology appears sequentially in interconnected brain regions originating from the injection site, and do not appear at early time points in regions that are not connected to the OB. It is also possible that PFFs injections trigger cellular dysfunction mechanisms that are transmitted to neighbouring neurons, instead of a transmission of α-syn seeds themselves; further work is needed to rule out this possibility. Finally, it is possible that cells that are able to migrate, such as microglia, might carry pathogenic seeds to distant brain regions, which has been suggested to occur in models of tauopathy [1]. However, in our model, we were unable to detect pser129 α-syn in microglia [59]; and if migrating cells were the vector of pathology spreading, we would expect appearance of pathology inversely proportional to distance from the injection site, and in regions that are not always directly and indirectly connected to the OB.

### Decrease in the density of α-syn pathology 18-month post-injection

Although pathology progressed to additional brain regions at 18-month post-injection, the overall density of pathology in the brain decreased. While mPFFs triggered dense-to-severe pathology in several brain regions at 12-month post-injection [59], only mild-to-moderate scores were observed at 18-month post-injection. Densitometry of pser129 pathology in olfactory regions confirmed a marked decrease in pathology density between 12- and 18-month post-injection in the OB, the PC, and the Ent after injection of mPFFs. In mice injected with huPFFs, moderate pathology was observed up to 18-month post-injection (confirmed with pser129 quantifications), while at 23 months, only mild pathology was present. Our observation of a decrease in the density of the pathology at later time points is consistent with the previous work. In a model of PFF-induced α-syn pathology by injection of PFFs into the striatum of rats, Paumier and colleagues described a decrease in pathology in the SN 180-day post-injection compared to earlier time points [49].

In our model, the pathology observed between 1 and 12-month post-injection was relatively severe in olfactory structures, and particularly after mPFFs injections [59]. We believe that neurons which contained pathology [47] before 12 months died, and were then degraded which resulted in an apparent decrease in the quantity of pser129-positive aggregates at 18 months.

Our data show that the injection of PFFs in WT mice induces α-syn pathology that progresses further in terms of spatial spreading until 18-month post-injection, but that the density of α-syn aggregate pathology does not increase after more than a year and declines later (the last time point studied was 18 months for mPFFs, and 23 months for huPFFs). Thus, to reproduce more closely the long-term rise in the severity of pathology in mouse models, as observed at late stages of PD, additional treatments beyond single PFFs injections appear to be necessary. Other unknown factors may contribute to the induction of α-syn pathology following injections of α-syn PFFs and they might be necessary to model the aging state of the human brain when sporadic PD pathology develops. For example, aged mice or those that display defects in protein clearance or in mitochondrial activity, or senescence accelerated-mice could be used.

### Severe cell loss in the AON, but not the OB, following PFFs injections

In the same model of progressive α-synucleinopathy that we describe in this paper, we have previously reported olfactory deficits which progressively develop over 1–12 months [59]. We also previously described that there was no loss of mitral cells in the OB 6 months after injection of PFFs [59]. To determine if mitral cells undergo delayed degeneration in the OB, we assessed whether injection of PFFs led to a loss of neurons after 18 months. We found no decrease in mitral cells density at 18-month post-injection, indicating that mitral cells are resistant to PFF-induced insults despite the injection being made directly into the OB and these cells being the first cells affected by the pathogenic α-syn fibrils [59].

We next examined if there was cell loss in the AON that could potentially contribute to the olfactory deficits which we previously reported [59]. A shortcoming with our approach when we quantified the numbers of cresyl violet-stained cells in the AON is that we could not distinguish small neurons from glia. While cresyl violet-positive large pyramidal neurons of the AON can easily be identified by morphology (cresyl violet-positive, large faint cells that we quantified), our approach does not allow us to differentiate glial cells from small interneurons or small pyramidal neurons that exhibit similar morphologies (cresyl violet-positive, small dark cells). However, statistical analyses demonstrate that the large faintly cresyl violet-positive cells (large pyramidal neurons) and the small dark cresyl violet-positive cells (mix of glial cells and small neurons) are affected by the injectate and by aging in a similar way.

Our stereological counting in the AON revealed severe loss of cresyl violet-positive large pyramidal neurons 6 months after injection of huPFFs and mPFFs. In the AON, 40–60% of the cresyl violet-stained cells disappeared, a magnitude in the same range as the loss of AON neurons observed in PD patients [50]. At 18-month post-injection, we observed a decrease in AON cell number in control animals (including in age-matched non-injected animals) compared to young mice, indicating an effect of normal aging. No further cell loss was observed in PFFs injected animals at 18 months. PFFs injection precipitates cell loss in the AON by 12 months. Data on loss of cells in the AON during aging are sparse as one study demonstrates a decrease of neuropeptide Y neurons with age [25]. The lack of further cell loss in the AON at 18-month post-injection suggests that a specific cell population in the AON was affected at 6 months, and that this same population eventually succumbs as a result of normal aging in our control mice. A recent study suggests that α-syn PFFs can trigger mitochondrial dysfunction in a specific subpopulation of cultured cells [68], and it is conceivable that a similar mechanism contributes to neuronal death in our in vivo paradigm. Further studies are required to precisely define which neurons die, and to what extent different neuronal subtypes (e.g., interneurons versus projection neurons) are selectively vulnerable in the AON and other olfactory regions in this mouse model. Finally, studies that define the neuroinflammatory response are needed to determine its contribution to propagation of α-syn aggregates and cell loss.

## Conclusions

We demonstrate that injection of α-syn PFFs into OB triggered progressively increasing α-syn pathology, followed by severe cell loss at 6-months post-injection and that pathology decreased in severity with slight anatomical progression at 18 months in our model. The former indicates that at early time points, our model of prodromal PD can be used to identify compounds that halt the propagation of α-syn pathology and prevent cell loss. Altogether, our previous work and present work demonstrate that our paradigm is suited for modelling olfactory-related pathology at prodromal stages between 1 and 12 months. Further studies are needed to explore the long-term effect of injections of α-syn PFFs in mice with alterations that mimic those associated with increased PD risk (e.g., mitochondrial deficits and neuroinflammation).

## Electronic supplementary material

Below is the link to the electronic supplementary material.

Supplementary material 1 (PDF 21 kb)

Supplementary material 2 (PDF 553 kb)

Supplementary material 3 (PDF 797 kb)

Supplementary material 4 (PDF 35 kb)

Supplementary material 5 (PDF 88 kb)

Supplementary material 6 (PDF 74 kb)

## Abbreviations

AON: Anterior olfactory nucleus
α-syn: Alpha-synuclein
BLA, BLP, BLV: Basolateral amygdala
CeL, CeL, CeC, CeA: Central amygdaloid nucleus of the amygdala
DP: Dorsal peduncular cortex
Ect: Ectorhinal cortex
Ent: Entorhinal cortex
Hipp: Hippocampus
huPFFs: Pre-formed fibrils of recombinant human α-synuclein
IL: Infra-limbic cortex
Ins: Insular cortex
LC: Locus coeruleus
LH: Lateral hypothalamic area
M1, M2: Primary and secondary motor cortex
mMs: Mouse monomeric α-synuclein
mPFFs: Pre-formed fibrils of recombinant mouse α-synuclein
OB: Olfactory bulb
PC: Piriform cortex
PD: Parkinson’s disease
PLH: Peduncular part of the lateral hypothalamic area
PFFs: Pre-formed fibrillar assemblies of recombinant α-synuclein
Pser129: α-Syn phosphorylated on serine 129
PVA: Paraventricular thalamic nucleus, anterior part
RML: Retro mammillary nucleus, lateral part
RN: Raphe nucleus
S1, S2: Primary and secondary somatosensory cortices
SN: Substantia nigra
TeA: Association area of the temporal cortex
V2: Secondary visual cortex
