# Supplementary material for: Spread of aggregates after olfactory bulb injection of α-synuclein fibrils is associated with early neuronal loss and is reduced long term
Source: Acta Neuropathol. 2017 Dec 5;135(1):65–83. doi: 10.1007/s00401-017-1792-9 (PMC5756266; doi:10.1007/s00401-017-1792-9)
Supplement: Supplementary file 1 — Supplementary material 1 (PDF 21 kb) [file 401_2017_1792_MOESM1_ESM.pdf]

**Online resource 1: Animals used in the current study**

The mice used in this study were part of 4 experimental replicates used in Rey et al. 2016.
The mice that were not euthanized for histological analyses in the previous study were maintained alive for up to 23 months post injection, and used for this study.
In addition, we reassessed some animals that were used in our previous study (for the experiments indicated in the table), and included an additional group, 9 months post injection, that we did not presented previously.

| | | | Animals | Animals used in Rey et al. 2016 | | Figures including these animals |
|---|---|---|---|---|---|---|
| **Timepoint** | **Experimental group** | **n=** | | **Reused for heatmap** | **Reused for quantifications** | |
| 1 mo | Ctl | 4 | Revisited | Simple scoring, calculation of average score value (continuous data) | | Fig 4 |
| | PBS | 4 | | | | |
| | mMs | 3 | | | | |
| | mPFFs | 4 | | | | |
| | huPFFs | 4 | | | | |
| 3 mo | Ctl | 3 | Revisited | Simple scoring, calculation of average score value (continuous data) | | Fig 4 |
| | PBS | 4 | | | | |
| | mMs | 5 | | | | |
| | mPFFs | 4 | | | | |
| | huPFFs | 3 | | | | |
| 6 mo | Ctl | 4 | Revisited | Simple scoring, calculation of average score value (continuous data) | New cresyl violet staining and stereology quantification | Fig 4, Fig 6 |
| | PBS | 4 | | | | |
| | mMs | 5 | | | | |
| | mPFFs | 5 | | | | |
| | huPFFs | 4 | | | | |
| 9 mo | Ctl | - | **New** | | | Fig 4 |
| | PBS | 4 | | | | |
| | mMs | 5 | | | | |
| | mPFFs | 5 | | | | |
| | huPFFs | 5 | | | | |
| 12 mo | Ctl | 4 | Revisited | Simple scoring, calculation of average score value (continuous data) | Pser129 slides from Rey et al. newly analysed by ImageJ for quantifications | Fig 4, Fig 5 |
| | PBS | 4 | | | | |
| | mMs | 9 | | | | |
| | mPFFs | 5 | | | | |
| | huPFFs | 5 | | | | |
| 18 mo | Ctl | 3 | **New** | | | Fig 1, Fig 2, Fig 3, Fig 4, Fig 5, Fig 6, Fig 7, Online resource 3 |
| | PBS | 3 | | | | |
| | mMs | 4 | | | | |
| | mPFFs | 3 | | | | |
| | huPFFs | 5 | | | | |
| 23 mo | Ctl | 3 | **New** | | | Fig 1, Fig 3, Fig 4 |
| | PBS | - | | | | |
| | mMs | 4 | | | | |
| | mPFFs | - | | | | |
| | huPFFs | 4 | | | | |
