# Supplementary material for: Spread of aggregates after olfactory bulb injection of α-synuclein fibrils is associated with early neuronal loss and is reduced long term
Source: Acta Neuropathol. 2017 Dec 5;135(1):65–83. doi: 10.1007/s00401-017-1792-9 (PMC5756266; doi:10.1007/s00401-017-1792-9)

Online resource 2 : Flow-chart of macros for pser129 analysis by ImageJ

Images were acquired at 20x magnification with condenser on for OB (a), and without condenser for other brain regions (b). Images were then processed on ImageJ64 as described in a and b.

a. Analysis of OB images

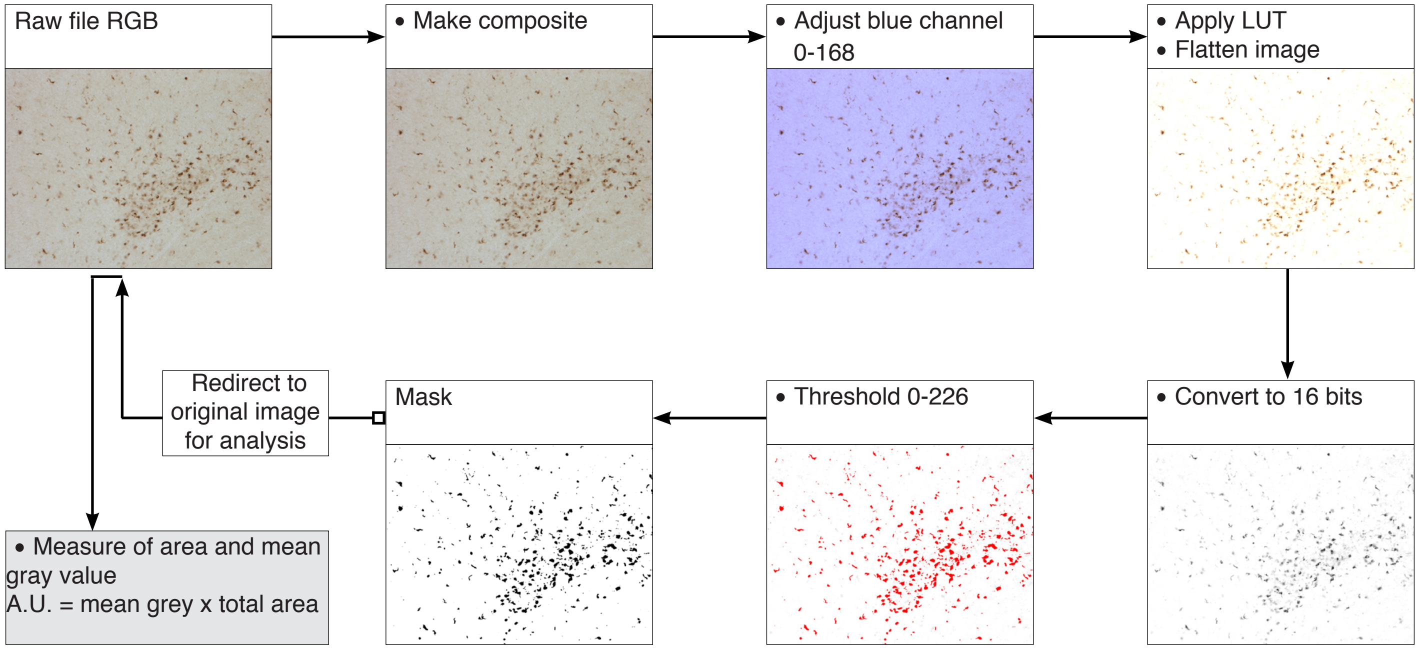

b. Analysis of images from other brain regions

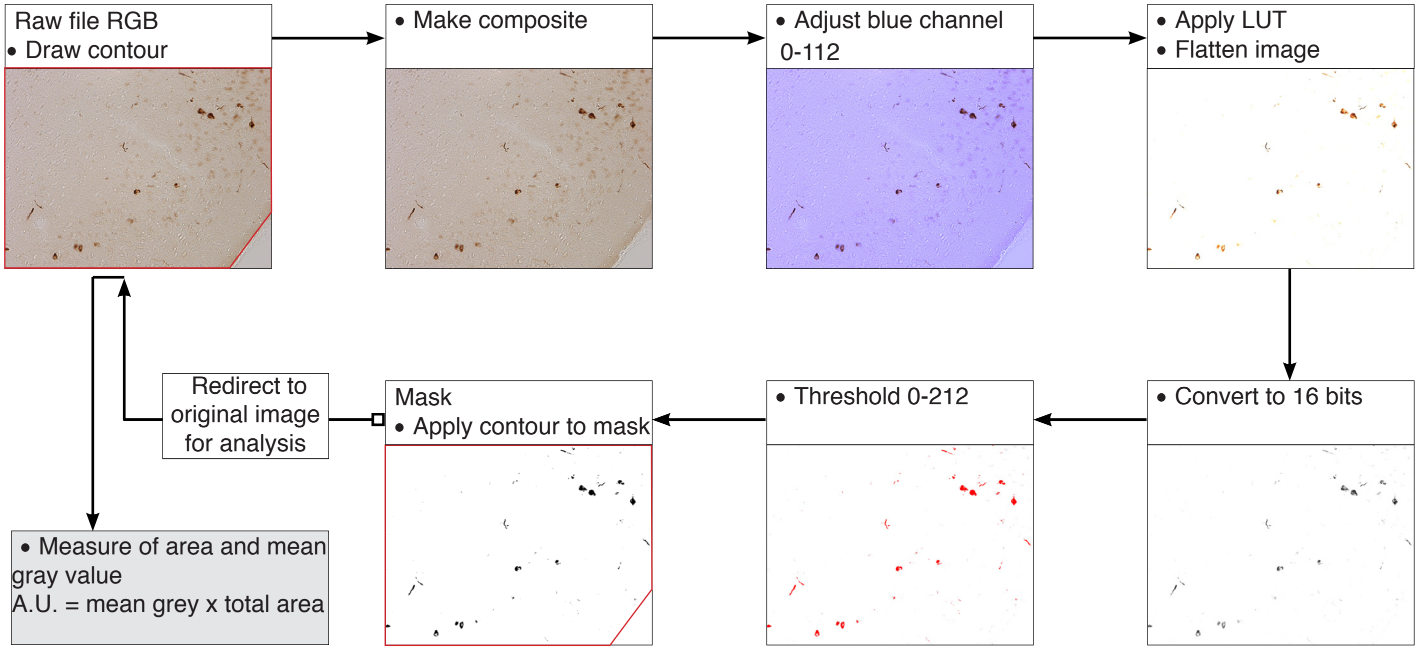

Supplement: Supplementary file 2 — Supplementary material 2 (PDF 553 kb) [file 401_2017_1792_MOESM2_ESM.pdf]
