# Supplementary material for: Spread of aggregates after olfactory bulb injection of α-synuclein fibrils is associated with early neuronal loss and is reduced long term
Source: Acta Neuropathol. 2017 Dec 5;135(1):65–83. doi: 10.1007/s00401-017-1792-9 (PMC5756266; doi:10.1007/s00401-017-1792-9)

Online resource 3 : Pser129 staining does not lead to unspecific staining in white matter tracts in control animals, and no TDP-43 or Tau pathologies are observed in PFFs injected mice.
(a) Pser129 staining is not detected in white matter tracts (aca: anterior part of the anterior commissure, fmi: anterior forceps of corpus callosum, cc: corpus callosum, and cerebellum) of mice injected with mMs 18 months-post injection (ipsilateral) and in age-matched non-injected mice (Ctl). Analysis was performed in 3 animals per group. Scale bar: 100 μm (b) Tau and TDP-43 pathologies were assessed by immunohistochemistry against hyperphosphorylated tau (AT8) (pS202/T205) and hyperphosphorylated TDP-43 (1D3) (pS409/410) (b). Tau and TDP-43 pathologies are detected respectively in post-mortem brain tissue from patients with AD (AD Brain, angular gyrus) and FTLD (FTLD Brain, cingulate cortex), but are absent from mice 18 months after injection of PBS, huPFFs or mPFFs (a-b; images from the ipsilateral anterior olfactory nucleus). Histochemical analysis was performed in the ipsilateral olfactory bulb and ipsilateral anterior olfactory nucleus of mice 18-months post-injection of mPFFs, HuPFFs and PBS (mPFFs 18 months: n=3; HuPFFs 18 months: n=5, PBS 18 months: n=1). Additional brain regions were assessed in one animal per group (hippocampus, orbitofrontal cortex, motor cortex, striatum, piriform cortex; data not shown). Scale bar: 20 μ m.

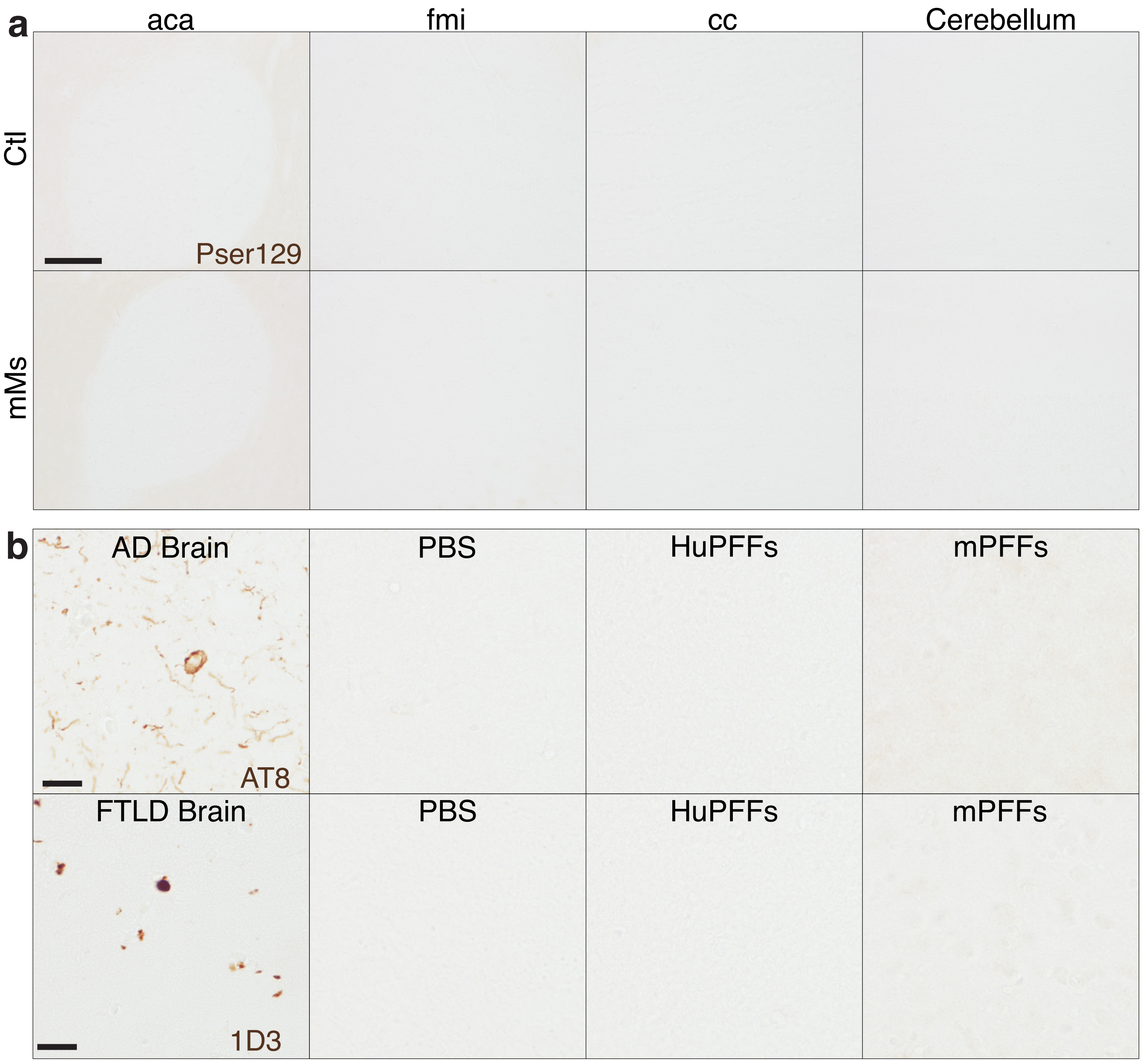

Supplement: Supplementary file 3 — Supplementary material 3 (PDF 797 kb) [file 401_2017_1792_MOESM3_ESM.pdf]
