# Supplementary material for: Spread of aggregates after olfactory bulb injection of α-synuclein fibrils is associated with early neuronal loss and is reduced long term
Source: Acta Neuropathol. 2017 Dec 5;135(1):65–83. doi: 10.1007/s00401-017-1792-9 (PMC5756266; doi:10.1007/s00401-017-1792-9)
Supplement: Supplementary file 4 — Supplementary material 4 (PDF 35 kb) [file 401_2017_1792_MOESM4_ESM.pdf]

Online resource 4

List of the abbreviations used in the figures

Abbreviation	Structure name
AA	Anterior amygdaloid area
AcbNu	Accumbens Nucleus Core and Shell
aca	Anterior part of the anterior commissure
aci	Anterior part of the anterior commissure, intrabulbar part
ACo	Anterior cortical nucleus of the amygdala
AHiPM, AHiAL	Amygdalohippocampal area posteromedial, part anterolateral part
APir	Amygdalopiriform transition area
AON	Anterior olfactory nucleus
AOB	Accessory olfactory bulb
Au2	Secondary auditory cortex
BAOT	Bed nucleus of accessory olfactory tract
BLA, BLV, BLP	Basal amygdaloid nucleus
BMA, BMP, BML	Accessory basal amygdaloid nucleus (Amygdala)
CA	Cornu Ammonis of the hippocampus
CeA	Central amygdaloid nucleus (Amygdala)
CeL, CeM, CeC	Central amygdaloid nucleus (Amygdala), Lateral, Medial, Central
Cg	Cingulate cortex
CGb	Central gray, beta part
Cp	Cerebral crus
CPu	Caudate putamen
CxA	Cortex amygdala transition area
DEn, IEn	Dorsal endopiriform nucleus, Immediate endopiriform Nucleus
DG	Dentate Gyrus
DMX	Dorsal motor nucleus of the vagus nerve
DP	Dorsal peduncular cortex
Ect	Ectorhinal cortex
Ent, DiEnt, MiEnt, CEnt, ViEnt	Entorhinal cortex
E/OV	Ependymal and subependymal layer/olfactory ventricle
FC	Frontal cortex and orbital cortex
HDB	Magnocellular preoptic nucleus
GP	Globus pallidus
Hipp	Hippocampus
IC	Internal capsule
Ins	Insular cortex
IL	Infra-limbic cortex
IPR	Interpeduncular nucleus
iRt, 7SH	Intermediate reticular nucleus; facial motor nucleus, stylohyoid part
LC	Locus Coeruleus
LH	Lateral hypothalamic area
LPO	Lateral preoptic nucleus
LSI	Lateral septal nucleus
M2	Secondary motor cortex
MeAD, MePV	Medial Nucleus of the amygdala
Med	Medial cerebellar nucleus
mPtA	Medial parietal association cortex
Mol	Molecular layer of the hippocampus
MoDG	Molecular layer, dentate gyrus
mVeMC, mVePC	Medial vestibular nucleus, magnocellular part, parvicellular part
nLOT	Nucleus of the lateral olfactory tract
OB	Olfactory bulb
Or	Oriens layer of the hippocampus
OT	Olfactory tubercle
PBP	Parabrachial pigmented nucleus
PC	Piriform cortex
PLCo	Posterolateral cortical amygdaloid area
PLH	Peduncular part of the lateral hypothalamic area

PMCo	Posteromedial cortical amygdaloid area
PoDG	Pyramidal layer, dentate gyrus
PRh	Perirhinal cortex
PVA	Paraventricular thalamic nucleus, anterior part
PVP	Paraventricular thalamic nucleus, posterior part
Pyr	Pyramidal layer of the hippocampus
REth	Retroethmoid nucleus
Rad	Radial layer of the hippocampus
rmx, RML	Retromammillary decussation, retromammillary nucleus, lateral part
RN	Dorsal and medial raphe nuclei
S1	Primary somatosensory cortex
S2	Secondary somatosensory cortex
SC	Superior colliculus
SFi	Septofimbrial nucleus
SN, SNR, SNpc	Substantia nigra, reticulata, pars compacta
STMAM, STMPM	Antero medial part of the bed nucleus of the stria terminalis
STr	Subiculum transition area
TeA	Temporal cortex association area
TT	Tenia tecta
V2	Secondary visual cortex
VS	Ventral subiculum
VP	Ventral pallidum
VTA	Ventral tegmental area
