# Supplementary material for: Spread of aggregates after olfactory bulb injection of α-synuclein fibrils is associated with early neuronal loss and is reduced long term
Source: Acta Neuropathol. 2017 Dec 5;135(1):65–83. doi: 10.1007/s00401-017-1792-9 (PMC5756266; doi:10.1007/s00401-017-1792-9)
Supplement: Supplementary file 5 — Supplementary material 5 (PDF 88 kb) [file 401_2017_1792_MOESM5_ESM.pdf]

Online resource 5: Linear mixed effect model analysis of pser129 quantifications

a. Comparison of ipsilateral versus contralateral sides, within the same experimental groups and delays post-injection.

Analyses for each brain regions were performed separately, but are presented in the same table for easier reading. ^ p<0.05, ^^ p<0.01, ^^^ p< 0.001. M. estim. = Model estimate

Brain region	Linear mixed effect model	12 mo			18 mo		
		mMs	HuPFFs	mPFFs	mMs	HuPFFs	mPFFs
OB	M. Estim.	-0.2415421	-2.1282153	-2.4606963	-0.0988964	-1.4449104	2.5365672
	SE	0.6318344	0.6318344	0.7064124	0.7064124	0.6318344	0.8156947
	p-value	0.8426982	0.0022689 ^^	0.0022689 ^^	0.8886615	0.0333069 ^	0.0037455 ^^
AON	M. Estim.	0.2698275	-1.4503223	-1.0137230	-0.4571813	-1.6265008	-0.7691579
	SE	0.4722032	0.4722032	0.4722032	0.5279392	0.4722032	0.6096117
	p-value	0.5677133	0.0063918 ^^	0.0636193	0.4638057	0.0034328 ^^	0.3105755
aPC	M. Estim.	0.1276413	-1.8216912	-1.7025134	0.8088221	-2.0827230	-2.7913161
	SE	0.3923716	0.3923716	0.3923716	0.5065496	0.4530717	0.5849131
	p-value	0.7449487	0.0000086 ^^^	0.0000215 ^^^	0.1323915	0.0000086 ^^^	0.0000086b ^^^
pPC	M. Estim.	0.2237275	-2.1453953	-1.8106423	-0.6666596	-3.6460458	-1.8887278
	SE	0.5569773	0.5569773	0.5569773	0.6227195	0.5569773	0.7190546
	p-value	0.6879184	0.0003517 ^^^	0.0023013 ^^	0.3412399	0.0000000 ^^^	0.0129333 ^
PC	M. Estim.	0.1813210	-1.9837258	-1.7828835	0.0710812	-2.8643844	-2.3400220
	SE	0.3162032	0.3162032	0.3162032	0.3872683	0.3463833	0.4471789
	p-value	0.6796228	0.0000000 ^^^	0.0000000 ^^^	0.8543703	0.0000000 ^^^	0.0000003 ^^^
Ent	M. Estim.	0.0461447	-2.3131974	-1.5910613	-0.0927822	-2.6742402	-3.0255354
	SE	0.4498931	0.4498931	0.5029958	0.5029958	0.4498931	0.5808095
	p-value	0.9183058	0.0000005 ^^^	0.0023409 ^^	0.9183058	0.0000000 ^^^	0.0000005 ^^^

b. Comparison between experimental groups, within same side of the brain and same delay. Analyses for each brain regions were performed separately, but are presented in the same table for easier reading. * p<0.05, ** p<0.01, *** p<0.001 for comparisons to mMs; # p<0.05, ## p<0.01, ### p<0.001 for comparisons between mPFFs and huPFFs. M. estim. = Model estimate

Brain region	Side	Linear mixed effect model	12 mo			18 mo		
			huPFFs/ mMs	mPFFs/mMs	huPFFs/mPFFs	huPFFs/ mMs	mPFFs/mMs	huPFFs/mPFFs
OB	Ipsi-lateral	M. estim.	4.9647172	-6.9911420	-2.0264248	2.4860425	-0.0516132	2.4344292
		SE	0.7505855	0.7961162	0.7961162	0.7961162	0.9064182	0.8667015
		p-value	0.0000000 ***	0.0000000 ***	0.0130988 #	0.0035838 **	0.9545914	0.007458 ##
	Contra-lateral	M. estim.	3.0780440	-4.7719880	-1.6939440	1.1400280	-2.6870770	-1.5470480
		SE	0.7505855	0.7961162	0.7961162	0.7961162	0.9064182	0.8667015
		p-value	0.0001235 ***	0.0000000 ***	0.0500355	0.1521474	0.0060635 **	0.0891167
AON	Ipsi-lateral	M. estim.	4.6859919	-5.9153911	-1.2293992	3.0398960	-2.3964439	0.6434521
		SE	0.5596034	0.5596034	0.5596034	0.5935490	0.6757853	0.6461743
		p-value	0.0000000 ***	0.0000000 ***	0.0336321 #	0.0000006 ***	0.0005863 ***	0.3193536
	Contra-lateral	M. estim.	2.9658421	-4.6318407	-1.6659985	1.8705765	-2.0844673	-0.2138908
		SE	0.5596034	0.5596034	0.5596034	0.5935490	0.6757853	0.6461743
		p-value	0.0000003 ***	0.0000000 ***	0.0034918 ##	0.0030583 **	0.0030583 **	0.7406362
aPC	Ipsi-lateral	M. estim.	2.7881870	-4.4508940	-1.6627070	3.1702950	-2.8222260	0.3480690
		SE	0.3870358	0.3870358	0.3870358	0.5329051	0.6067392	0.5801536
		p-value	0.0000000 ***	0.0000000 ***	0.0000209 ###	0.0000000 ***	0.0000049 ***	0.5485328
	Contra-lateral	M. estim.	0.8388548	-2.6207396	-1.7818848	0.2787496	0.7779126	1.0566622
		SE	0.5024278	0.5024278	0.5024278	0.5329051	0.6067392	0.5801536
		p-value	0.1424970	0.0000011 ***	0.0011709 ##	0.6009218	0.2397606	0.1371077
pPC	Ipsi-lateral	M. estim.	2.7532726	-4.2592406	-1.5059680	3.6456037	-3.0675061	0.5780976
		SE	0.5569773	0.5569773	0.5569773	0.5907636	0.6726140	0.6431420
		p-value	0.0000015 ***	0.0000000 ***	0.0082256 ##	0.0000000 ***	0.0000077 ***	0.3687247
	Contra-lateral	M. estim.	0.3841498	-2.2248709	-1.8407210	0.6662176	-1.8454379	-1.1792204
		SE	0.5569773	0.5569773	0.5569773	0.5907636	0.6726140	0.6431420
		p-value	0.4903799	0.0003889 **	0.0028509 ##	0.3113244	0.0121509 ***	0.1000856
PC	Ipsi-lateral	M. estim.	2.7765490	-4.3870097	-1.6104606	3.4079492	-2.9448659	0.4630833
		SE	0.3883009	0.3883009	0.3883009	0.4632686	0.4987108	0.4742004
		p-value	0.0000000 ***	0.0000000 ***	0.0000403 ###	0.0000000 ***	0.0000000 ***	0.3287889
	Contra-lateral	M. estim.	0.6115023	-2.4228052	-1.8113029	0.4724836	-0.5337627	-0.0612791
		SE	0.4367738	0.4367738	0.4367738	0.4632686	0.4987108	0.4742004
		p-value	0.3230007	0.0000002 ***	0.0001011 ###	0.3693361	0.3693361	0.8971787
Ent	Ipsi-lateral	M. estim.	4.3141972	-7.2342055	-2.9200083	4.8852957	-4.2210985	0.6641973
		SE	0.5303450	0.5625158	0.5625158	0.5625158	0.6404524	0.6123896
		p-value	0.0000000 ***	0.0000000 ***	0.0000003 ###	0.0000000 ***	0.0000000 ***	0.2780992
	Contra-lateral	M. estim.	1.9548550	-5.5969990	-3.6421440	2.3038380	-1.2883450	1.0154920
		SE	0.5303450	0.5625158	0.5625158	0.5625158	0.6404524	0.6123896
		p-value	0.0003417 ***	0.0000000 ***	0.0000000 ###	0.0000842 ***	0.0531123	0.0972679

c. Comparison of 12 mo versus 18 mo delays, within same experimental groups and same side of the brain.

Analyses for each brain regions were performed separately, but are presented in the same table for easier reading. $ p<0.05, $$ p<0.01, $$$ p<0.001. M. estimate= Model estimate

Brain region	Side	Linear mixed effect model	mMs	huPFFs	mPFFs
OB	Ipsi-lateral	M. estim.	-1.2572934	1.2213813	5.6822353
		SE	0.7961162	0.7505855	0.9064182
		p-value	0.1714064	0.1714064	0.0000000 $$$
	Contra-lateral	M. estim.	-1.3999392	0.5380763	0.6849719
		SE	0.7961162	0.7505855	0.9064182
		p-value	0.1714064	0.4734510	0.4734510
AON	Ipsi-lateral	M. estim.	-2.3096783	-0.6635824	1.2092689
		SE	0.5935490	0.5596034	0.6461743
		p-value	0.0005982 $$$	0.2828375	0.1225715
	Contra-lateral	M. estim.	-1.5826695	-0.4874039	0.9647038
		SE	0.5935490	0.5596034	0.6461743
		p-value	0.022997 $	0.3837645	0.2031767
aPC	Ipsi-lateral	M. estim.	1.2148021	0.8326947	2.8434706
		SE	0.4823581	0.4484587	0.5340971
		p-value	0.0235735 $	0.0760088	0.0000003 $$$
	Contra-lateral	M. estim.	0.5336212	1.0937265	3.9322734
		SE	0.5329051	0.5024278	0.5801536
		p-value	0.3166606	0.0442339 $	0.0000000 $$$
pPC	Ipsi-lateral	M. estim.	0.5282631	-0.3640680	1.7199976
		SE	0.5907636	0.5569773	0.6431420
		p-value	0.4454554	0.5133375	0.0224611 $
	Contra-lateral	M. estim.	1.4186502	1.1365825	1.7980831
		SE	0.5907636	0.5569773	0.6431420
		p-value	0.0326664 $	0.0619320	0.0224611
PC	Ipsi-lateral	M. estim.	0.8658960	0.2344958	2.3080398
		SE	0.4411586	0.4132487	0.4526247
		p-value	0.0596065	0.5704120	0.000001 $$$
	Contra-lateral	M. estim.	0.9761357	1.1151545	2.8651783
		SE	0.4632686	0.4367738	0.4742004
		p-value	0.0526683	0.02135 $	0.0000000 $$$
Ent	Ipsi-lateral	M. estim.	0.8401741	0.2690756	3.8532811
		SE	0.5625158	0.5303450	0.6404524
		p-value	0.2029200	0.6119026	0.0000000 $$$
	Contra-lateral	M. estim.	0.9791010	0.6301184	5.2877552
		SE	0.5625158	0.5303450	0.6404524
		p-value	0.1635162	0.2817390	0.0000000 $$$

d. Comparison between quantifications in aPC and pPC

	**aPC vs pPC**
M. estim.	0.0426258
SE	0.1344211
p-value	0.7511627
