# Supplementary material for: Spread of aggregates after olfactory bulb injection of α-synuclein fibrils is associated with early neuronal loss and is reduced long term
Source: Acta Neuropathol. 2017 Dec 5;135(1):65–83. doi: 10.1007/s00401-017-1792-9 (PMC5756266; doi:10.1007/s00401-017-1792-9)
Supplement: Supplementary file 6 — Supplementary material 6 (PDF 74 kb) [file 401_2017_1792_MOESM6_ESM.pdf]

Online resource 6: Linear mixed effect model analysis of cresyl-positive cells quantifications

a. Comparison of ipsilateral versus contralateral sides for each experimental group and delay.
^ p<0.05, ^^ p<0.01, ^^^ p< 0.001. M. estim. = Model estimate

Delay	Cell type	Linear mixed effect model	Ctl	mMs	HuPFFs	mPFFs
6 mo	All	M. Estim.	0.1273718	0.0932906	-0.0660213	-0.0457503
		SE	0.1048393	0.0813596	0.0910254	0.0814151
		p-value	0.6707416	0.6707416	0.9186515	0.9186515
	Dark stained	M. Estim.	0.1303246	0.1123022	-0.1992801	-0.0124842
		SE	0.1390759	0.1078551	0.1210392	0.1081838
		p-value	0.9081295	0.9081295	0.7974361	0.9081295
	Light stained	M. Estim.	0.1273563	0.0908026	0.0487940	-0.0652603
		SE	0.1518321	0.1179213	0.1318078	0.1178177
		p-value	0.7710894	0.7710894	0.8128464	0.7728550
18 mo	All	M. Estim.	0.0233055	0.1347899	0.0048061	-0.0301928
		SE	0.1049368	0.0913747	0.0822450	0.1049764
		p-value	0.9419926	0.6707416	0.9534006	0.9419926
	Dark stained	M. Estim.	-0.0645168	0.0877502	0.0236020	-0.0544401
		SE	0.1391695	0.1223140	0.1104617	0.1394721
		p-value	0.9081295	0.9081295	0.9081295	0.9081295
	Light stained	M. Estim.	0.1069331	0.1256423	0.5736342	-0.0139157
		SE	0.1520664	0.1319961	0.1206111	0.1519505
		p-value	0.7710894	0.7710894	0.0000158 ^^^	0.9270315

b. **Comparison between experimental groups within same side of the AON and the same delay.**

* p<0.05, ** p<0.01, *** p<0.001 for comparisons to mMs and to Ctl; # p<0.05, ## p<0.01, ### p<0.001 for comparisons between mPFFs and huPFFs. M. estim. = Model estimate

Delay	Cell type	Side	Linear mixed effect model	Ctl/ mMs	Ctl/ huPFFs	Ctl / mPFFs	mMs/ huPFFs	mMs/ mPFFs	huPFFs/ mPFFs
6 mo	All cells	Ipsi-lateral	M. Estim.	0.0317883	0.8894865	0.5869463	-0.8576983	0.5551580	-0.3025402
			SE	0.1845051	0.1930231	0.1845464	0.1695634	0.1598474	0.1696043
			p-value	0.8632101	0.0000244 ***	0.0044109 **	0.0000051 ***	0.0020583 **	0.1276390
		Contra-lateral	M. Estim.	0.0658694	1.0828796	0.7600685	-1.0170102	0.6941990	-0.3228112
			SE	0.1844927	0.1929744	0.1845418	0.1695465	0.1598824	0.1695989
			p-value	0.7210693	0.0000001 ***	0.0001143 ***	0.0000000 ***	0.0000565 ***	0.1139819
	Dark stained	Ipsi-lateral	M. Estim.	-0.0622179	0.8512679	0.4428020	-0.9134858	0.5050199	-0.4084659
			SE	0.3086262	0.3229035	0.3087010	0.2831354	0.2668252	0.2832155
			p-value	0.8402323	0.0502905	0.2596405	0.0150471 *	0.1401547	0.2596405
		Contra-lateral	M. Estim.	-0.0441954	1.1808727	0.5856109	-1.2250681	0.6298063	-0.5952618
			SE	0.3085971	0.3228471	0.3086641	0.2831266	0.2668408	0.2831988
			p-value	0.9047783	0.001527 **	0.0990786	0.0001814 ***	0.0730540	0.0990786
	Light stained	Ipsi-lateral	M. Estim.	0.1177756	1.0155376	0.6841849	-0.8977620	0.5664093	-0.3313527
			SE	0.1864471	0.1950463	0.1865344	0.1713428	0.1615703	0.1714054
			p-value	0.5755566	0.0000012 ***	0.000587 ***	0.0000012 ***	0.000911 ***	0.0798263
		Contra-lateral	M. Estim.	0.1543293	1.0940999	0.8768015	-0.9397706	0.7224722	-0.2172985
			SE	0.1863414	0.1948616	0.1865573	0.1711917	0.1616636	0.1714330
			p-value	0.5434042	0.0000002 ***	0.0000104 ***	0.0000002 ***	0.0000236 ***	0.4099234
18 mo	All cells	Ipsi-lateral	M. Estim.	0.4599798	0.3981126	0.1454395	0.0618672	-0.3145402	-0.2526730
			SE	0.1930503	0.1846292	0.2063084	0.1697449	0.1931022	0.1846836
			p-value	0.0412475 *	0.0621234	0.5770008	0.7805517	0.1550077	0.2283578
		Contra-lateral	M. Estim.	0.3484953	0.4166119	0.1989378	-0.0681166	-0.1495575	-0.2176740
			SE	0.1930554	0.1846798	0.2063090	0.1698052	0.1931074	0.1847353
			p-value	0.1218002	0.0577905	0.4465465	0.7210693	0.5263778	0.3580141
	Dark stained	Ipsi-lateral	M. Estim.	0.7901024	0.6920332	0.3837566	0.0980692	-0.4063458	-0.3082766
			SE	0.3229044	0.3088852	0.3449538	0.2834357	0.3223640	0.3083203
			p-value	0.0576425	0.0751906	0.3545718	0.7956454	0.3112235	0.3808550
		Contra-lateral	M. Estim.	0.6378355	0.6039144	0.3736800	0.0339210	-0.2641555	-0.2302345
			SE	0.3230084	0.3088963	0.3449653	0.2835555	0.3224726	0.3083361
			p-value	0.0990786	0.0990786	0.4180533	0.9047783	0.5462939	0.5462939
	Light stained	Ipsi-lateral	M. Estim.	0.2382181	0.7460466	-0.0315631	-0.5078285	-0.2697812	-0.7776097
			SE	0.1950609	0.1870274	0.2084143	0.1719291	0.1949954	0.1869467
			p-value	0.2663896	0.0001991 ***	0.8796255	0.0053825 **	0.2220048	0.0001276 ###
		Contra-lateral	M. Estim.	0.2195089	0.2793455	0.0892857	-0.0598366	-0.1302232	-0.1900598
			SE	0.1950501	0.1869135	0.2084113	0.1717607	0.1949313	0.1867961
			p-value	0.4464353	0.3240967	0.7275608	0.7275608	0.6049246	0.4633932

c. **Comparison of 6 months versus 18 months delays, within same experimental groups and same side of the brain.**

Analyses for each brain regions were performed separately, but are presented in the same table for easier reading. $ p<0.05, $$ p<0.01, $$$ p<0.001. M. estim. = Model estimate

Cell type	Side	Linear mixed effect model	Ctl	mMs	huPFFs	mPFFs
All cells	Ipsi-lateral	M. Estim.	0.6897681	1.1179596	0.1983941	0.2482613
		SE	0.1050046	0.1696019	0.1697091	0.1845937
		p-value	0.0000000 $$$	0.0000000 $$$	0.2770214	0.2766555
	Contra-lateral	M. Estim.	0.7938344	1.0764603	0.1275667	0.2327038
		SE	0.1049088	0.1696201	0.1697314	0.1846134
		p-value	0.0000000 $$$	0.0000000 $$$	0.4523038	0.2766555
Dark cells	Ipsi-lateral	M. Estim.	0.4596734	1.3119937	0.3004387	0.4006280
		SE	0.1392783	0.2831452	0.2834250	0.3081286
		p-value	0.001931 $$	0.0000096 $$$	0.3304358	0.2580444
	Contra-lateral	M. Estim.	0.6545148	1.3365457	0.0775566	0.4425839
		SE	0.1392119	0.2832711	0.2834119	0.3081401
		p-value	0.0000096 $$$	0.0000096 $$$	0.7843511	0.2414639
Light cells	Ipsi-lateral	Model estimate	0.8696058	0.9900483	0.6001149	0.1538579
		SE	0.1520737	0.1713408	0.1719170	0.1864867
		p-value	0.0000000 $$$	0.0000000 $$$	0.0007707 $$$	0.5458032
	Contra-lateral	M. Estim.	0.8900290	0.9552086	0.0752746	0.1025133
		SE	0.1518788	0.1712700	0.1716826	0.1865726
		p-value	0.0000000 $$$	0.0000000 $$$	0.6610584	0.6610584
